# Physiological and transcriptome analysis elucidates the metabolic mechanism of versatile *Porphyridium purpureum* under nitrogen deprivation for exopolysaccharides accumulation

**DOI:** 10.1186/s40643-021-00426-x

**Published:** 2021-08-12

**Authors:** Liang Ji, Shaohua Li, Cheng Chen, Haojie Jin, Haizhen Wu, Jianhua Fan

**Affiliations:** 1 State Key Laboratory of Bioreactor Engineering, East China University of Science and Technology, 130 Meilong Road, Shanghai, 200237 People’s Republic of China; 2 The College of Forestry, Beijing Forestry University, Beijing, 100083 People’s Republic of China; 3 Department of Applied Biology, East China University of Science and Technology, Shanghai, 200237 People’s Republic of China

**Keywords:** *Porphyridium purpureum*, Nitrogen deprivation, Transcriptome analysis, Exopolysaccharides, Polyunsaturated fatty acids, Phycoerythrin

## Abstract

*Porphyridium purpureum* is a mesophilic, unicellular red alga rich in phycoerythrin, sulfate polysaccharides, and polyunsaturated fatty acids. Nitrogen deficiency inhibited the growth of *P. purpureum* and resulted in yellowing of the cells and thickening of the extracellular viscousness sheath. Under nitrogen stress, the contents of total lipids and exopolysaccharides in *P. purpureum* were increased by 65.2% and 188.0%, respectively. We demonstrate that the immediate response of *P. purpureum* to nitrogen deficiency is mediated by carbon flow to polysaccharide synthesis, while the synthesis of lipids is enhanced as a permanent energy storage substance at the later stage. Based on transcriptome annotation information, we elucidate the synthesis pathway of polysaccharides from *P. purpureum* from the perspective of glycosyl-donor interconversion, and demonstrate that the n-6 pathway is the main synthesis pathway of polyunsaturated fatty acids. This study not only provides a production strategy for polysaccharides and fatty acids by single-celled marine red algae *P. purpureum*, but also provides targets for further genetic modification.

**Supplementary Information:**

The online version contains supplementary material available at 10.1186/s40643-021-00426-x.

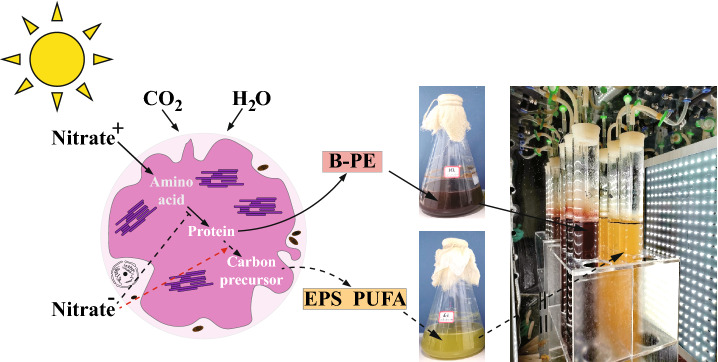

## Introduction

Ancestral lineages of red algae have acted as mediators between prokaryotes and photosynthetic eukaryotes and formed a monophyletic lineage that consists of several unicellular mesophilic and extremophilic species as well as economically important seaweeds, thereby significantly increasing the diversity of photosynthetic organisms. *Porphyridium purpureum* is a unicellular autotrophic marine red alga that is rich in phycoerythrin, polysaccharides, polyunsaturated fatty acids (PUFAs), and other high-value bioactive substances and has been widely used as food, healthcare products, and medical and aquatic products (Li et al. 2019). Like most microalgae, *P. purpureum* is characterized by fast growth, wide distribution, strong adaptability, and not competing for arable land (Hu et al. 2008).

Phycoerythrin is a bright red pigment with antioxidant activity and has been used as a coloring agent in the production of ice cream, candy, and other food items, as well as food additives (Kannaujiya and Sinha 2015). Recently, the structure and energy transfer mechanism of phycobilisome in *P. purpureum* have been established, and the results show that phycoerythrobilin and phycoerythrin are its main components and play a key role in energy transfer (Ma et al. 2020). *P. purpureum* exopolysaccharides are composed of xylose, galactose, glucose, and other monosaccharides and are rich in glucuronic acid and sulfate groups, which confers stronger antioxidant capacity (Sun et al. 2009). Arachidonic acid (ARA), a long-chain ω-6 PUFA, is important for brain and cardiovascular system development, especially for infant cognitive development, but the traditional deep-sea fish oil-derived ARA may be contaminated by heavy metals (Timilsena et al. 2017). Thus, a healthier and more sustainable source is needed. Actually, *P. purpureum* is one of the few microalgae that are rich in ARA, and the culture medium is free of heavy metals, making it a potential host for producing ARA (Khozin-Goldberg et al. 2011).

Abiotic stress is one of the strategies to improve microalgae productivity. Studies have shown that high light intensities promote lipid accumulation and increase the proportion of triacylglycerol (TAG) in microalgae (Kurpan Nogueira et al. 2015). Appropriate increases in temperature can promote lipid accumulation and improve neutral lipid content, whereas low temperature reduces PUFA content and membrane fluidity (Renaud et al. 2002). For nutrient stress, nitrogen and phosphorus stress has been widely used for metabolism regulation in microalgae (Jaeger et al. 2017; Liu et al. 2019; Miller et al. 2010; Siaut 2011). Transcriptome analysis has shown that *Chlamydomonas reinhardtii* reinforces the de novo synthesis of fatty acids and membrane lipid turnover to promote carbon flow to lipid accumulation under nitrogen deficiency (Miller et al. 2010). Studies combining transcriptome with lipidome analyses have shown that several metabolic pathways are stimulated to cope with nitrogen depletion in *Nannochloropsis oceanica* IMET1, which include upregulation of Kennedy pathway-related genes, utilization of both pyruvate dehydrogenase complex (PDHC) and PDHC bypass pathway, and enhancement of genes involved in tricarboxylic acid (TCA) cycle (Li et al. 2014). Multi-omics analysis of *Chromochloris zofingiensis* under nitrogen deprivation not only clarified the oleaginous mechanism but also revealed some key pathway-related genes, which provided targets for further genetic modification (Liu et al. 2019).

Although *P. purpureum* is a non-model organism, its genetic background is relatively clear, and its genome information has been published (Bhattacharya et al. 2013). Studies have shown that bacteria-derived plasmids could stably and freely exist in *P. purpureum*, and the expression level of foreign genes was as high as 5% of the total soluble protein (TSP) due to the high copy property of the plasmids, which not only provided a new method for genetic engineering research of *P. purpureum*, but also explored its potential as a bioreactor for the production of high bioactive substances (Li and Bock 2018). The stable nuclear transformation system of *P. purpureum* has also been established, which further enriched its genetic engineering research (Prasad et al. 2019).

However, due to the lack of suitable gene targets, culture optimization remains the major foci of current studies on *P. purpureum*, and its genetic engineering has not been reported until recently by a study showing that knock out of the chlorophyll synthase gene (*CHS1*) resulted in phycoerythrin production (Jeon et al. 2021). In the early stage, our laboratory conducted culture optimization and carbon-to-nitrogen ratio regulation on *P. purpureum*, and assessed differences in the physiological and biochemical components of *P. purpureum* under nitrogen-deficient conditions but did not elucidate its metabolic mechanism at the molecular level (Li et al. 2020). Unlike the well-studied mechanisms of nitrogen deficiency in green algae, *P. purpureum* acts as a mediator of horizontal gene transfer between prokaryotes and photosynthetic eukaryotes (Bhattacharya et al. 2013), which makes it significant to study the response mechanism of *P. purpureum* to nitrogen deprivation. In this study, we performed transcriptome analysis of *P. purpureum* under nitrogen deprivation for the first time. Synthesis pathways of polysaccharides and PUFAs and the metabolic mechanism underlying nitrogen deficiency were elucidated, and several key pathway-related genes were identified, providing targets for further rational genetic engineering. This study further lays a foundation for the accumulation of high-value bioactive substances from *P. purpureum* as a “green factory”.

## Materials and methods

### Strains and growth conditions

*P. purpureum* FACHB-806 was purchased from the Freshwater Algae Culture Collection at the Institute of Hydrobiology. The ASW medium previously optimized by our laboratory was used for culture (Li et al. 2020), and the culture temperature, rotation speed, and illumination conditions were 25 °C, 120 rpm, and 50 μmol/m^2^/s, respectively.

### Nitrogen deficiency experimental design

The KNO3 concentration in nitrogen-deficient ASW medium was 0.075 g/L (1.000 g/L in normal ASW medium), and other components were the same as those in normal ASW medium. Seed culture was conducted using a 1-L flask with 500 mL normal ASW medium. The algal cells in logarithmic phase were collected by centrifugation at 4000 rpm for 10 min, washed with sterile deionized water, and then resuspended in nitrogen-deficient and normal ASW medium. Approximately 500 mL of mixed algal cells was transferred to a vertical columnar photoreactor (6 × 60 cm) with an initial inoculation density of 2.0  ×  10^6^ cells/mL, and the culture temperature, aeration rate (sterile air), and illumination conditions were 25 °C, 1 vvm, and 50 μmol/m^2^/s, respectively.

Samples were taken every 2.5 days and 5 days for growth characteristics and biochemical components measurement, respectively. Algal cells were collected at 5 and 15 days for transcriptome analysis. Cells from day 12 were collected for scanning electron microscopy (SEM) and transmission electron microscopy (TEM).

### Physiological and biochemical analysis

For growth characteristics, cell density was measured by a hemocytometer, and dry cell weight was calculated as previous reported (Li et al. 2020). For chlorophyll *a* concentration determination, 2 mL algal cells (1.0 × 10^7^ cells/mL) were centrifuged, washed with sterile deionized water, and finally soaked in 90% acetone at 4 °C for 24 h in the dark. Then, the supernatant was collected (4 °C, 5000 rpm for 10 min), and the absorbance was determined with a spectrophotometer at a wavelength of 665 nm. Chlorophyll *a* concentration was further calculated as described elsewhere (Jia 2006). The OJIP test was measured with a portable fluorometer AquaPen-C 100 (Photon System Instrument, Czech) using the FluoPen 1.0 software to access the data (Li et al. 2018). Two milliliters of algal cells (1.0 × 10^7^ cells/mL) were collected and adapted to the dark for 20 min before measurement. For SEM and TEM (HITACHI SU9010, Japan), cell pretreatment and section preparation were performed according to standard procedures (Arsiya et al. 2017). Total soluble protein, carbohydrate, total lipid, polyunsaturated fatty acid, phycoerythrin, and exopolysaccharide content were determined as previously described (Li et al. 2020).

### RNA sequencing and data analysis

RNA samples from four growth phases (8 samples) were submitted to Beijing Biomarker Technologies Corporation for Illumina sequencing. The mRNA-seq paired-end sequencing data are available at the NCBI’s Sequence Read Archive database with an accession number PRJNA682303. Clean data (clean reads) were obtained by removing reads containing adapter, reads containing poly-Ns, and low-quality reads from raw data. At the same time, Q20, Q30, and GC content of the clean data were calculated. Clean reads were then mapped to the *P. purpureum* genome (GenBank Assembly Accession: GCA_008690995.1) using HISAT2 tools software (http://ccb.jhu.edu/software/hisat2/index.shtml) (Kim et al. 2015). The mapped reads were spliced using StringTie software (https://ccb.jhu.edu/software/stringtie/index.shtml) and compared with the reference genome annotation to discover new genes (Pertea et al. 2015). DEseq (http://www.bioconductor.org/packages/release/bioc/html/DESeq.html) was used for differential expression analysis (Wang et al. 2010), and the false discovery rate (FDR)  < 0.05 and |Log_2_FC| ≥ 1 were set as the threshold for significant differential expression. Gene function was annotated based on the Nr, Nt, Pfam, KOG/COG, Swiss-Prot, KO, and the GO databases using BLAST (http://blast.ncbi.nlm.nih.gov/Blast.cgi). Gene Ontology (GO) enrichment analysis of DEGs was implemented by the GOseq R packages based Wallenius non-central hyper-geometric distribution (Young et al. 2010). KOBAS software was used to test the statistical enrichment of differential expression genes in KEGG pathways (Mao et al. 2005). Carbohydrate-active enzyme prediction was conducted using CAZY (http://www.cazy.org/).

### Statistical analysis

Each experiment and each assay had three biological replicates and two technical replicates, respectively. The data were expressed as the mean and standard deviation (SD). For mRNA-sequencing, two separate biological replicates were analyzed in parallel. Student’s *t* test was used as implemented in the IBM SPSS Statistic 24 software. For all the data analysis, differences with a *P* value  <  0.05 were considered statistically significant.

## Results and discussion

### Under nitrogen stress, the color of *P. purpureum* turned yellow, photosynthesis decreased, the structure of phycobilisome was damaged, but the thickness of the extracellular viscousness sheath increased

**Fig. 1:**
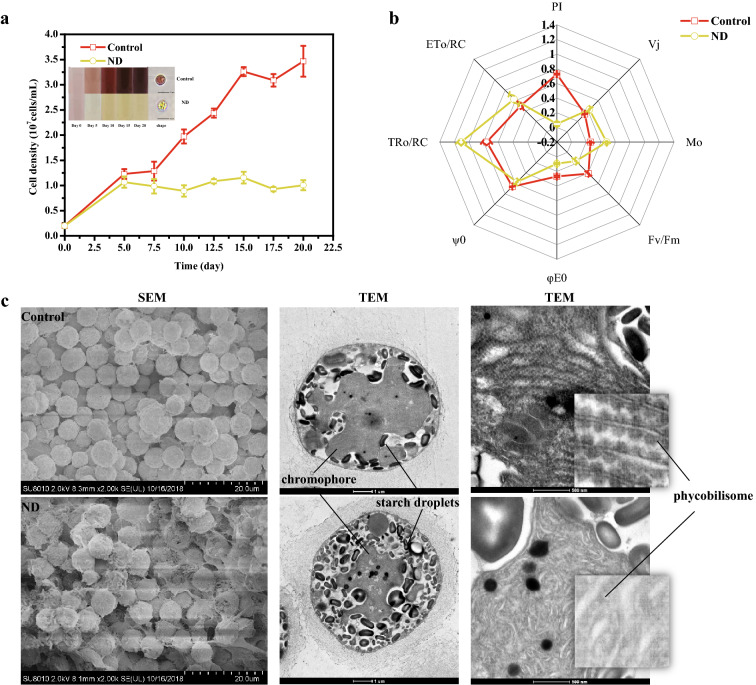
Growth characteristics, photosystem parameters and morphological changes of *P. purpureum* under normal and nitrogen-deficient conditions. **a** Growth curves and color changes of *P. purpureum* under normal and nitrogen-deficient conditions, and individual cell status under optical microscope at day 20. **b** Spider-plot presentation of selected parameters quantifying the behavior of PS II of *P. purpureum* cells exposed for 20 days to nitrogen stress. **c** Scanning electron microscope and transmission electron microscope images of normal and nitrogen-deficient cells on day 12. ND means nitrogen deprivation. Data are expressed as mean  ±  SD (*n*  =  3)

Owing to fluorescent phycoerythrin, *P. purpureum* exhibits a vivid red color under normal conditions, but when cultured under nitrogen deprivation conditions, the algae turned yellow by the fifth day (Fig. 1a), which is consistent with chlorosis in green algae under nitrogen deficiency (Arsiya et al. 2017; Li et al. 2020). Images from an optical microscope further confirmed the color changes of the single cell. *P. purpureum* under normal conditions rapidly grew, and cell density peaked at 3.46  ±  0.31  ×  10^7^ cells/mL on day 20, but cells grew slowly under nitrogen deprivation conditions and stopped growing on day 5, reaching a cell density of 1.07  ±  0.10  ×  10^7^ cells/mL (Fig. 1a).

Photosynthetic system (PS) plays an irreplaceable role in the development of microalgae. Fv/Fm, the maximum and effective quantum yields of PSII electron transport, showed a relatively flat trend under normal conditions, but significantly decreased under nitrogen deprivation conditions in *P. purpureum* (Additional file 1: Figure S1), as has been reported in other microalgae (Schmollinger et al. 2014). To further investigate the photosynthetic response of nitrogen deprivation, we ran the OJIP test on the cells under two different conditions on day 20. The results showed that the total energy absorption, trapping, and transfer (Fv/Fm, ψ0, and φE0, respectively) were much lower in ND cells (cells under nitrogen deprivation), leading to a drop in PI (Fig. 1b), which is an overall evaluation of photosynthetic efficiency (Aksmann and Tukaj 2008). However, the specific energy fluxes for absorption, trapping, and electron transport per reaction center (RC), namely, TR0/RC, ET0/RC, and ABS/RC, respectively, were higher in ND cells (Fig. 1b), indicating bigger size and stronger photosynthetic efficiency of individual RC (Aksmann and Tukaj 2008). This may be explained by the lower number of RCs in the cells under ND, which coincides with a decrease in the number of phycobilisome units observed by TEM (Fig. 1c).

Because exopolysaccharides (EPS) and phycobilisome are barely visible under the optical microscope, SEM and TEM were subsequently adopted (Fig. 1c). Both SEM and TEM showed that cells under ND tended to secrete more exopolysaccharides outside the cell, thereby resulting in the accumulation of cell wall polysaccharides (viscousness sheath), which facilitated intercellular contact. There was an intact star-shaped chromophore (chloroplast) within the CK cells (cells under normal conditions) of *P. purpureum*, but the chromophore under ND was much smaller, just about a third of the size of the CK cells. However, cell size did not significantly change in either conditions. Intracellular polysaccharides aggregated in the form of ‘starch granules’, which significantly accumulated in ND cells. We found the suspected phycobilisome structure inside the chromophore of CK cells, consistent to an earlier report (Ma et al. 2020), but was almost invisible in ND cells. Therefore, the lack of nitrogen not only caused shrinkage of the chromophores, but also reduced the content and stability of phycobilisomes in *P. purpureum*.

Taken together, nitrogen deficiency impaired the structure of chromophores and phycobilisomes, leading to a decrease in photosynthetic efficiency and further inhibition of *P. purpureum* growth. However, thickening of the extracellular viscousness sheath may be a protective mechanism under stress.

### Under nitrogen deficiency, the total soluble protein content decreased significantly, while the polysaccharide and lipid content increased

**Fig. 2:**
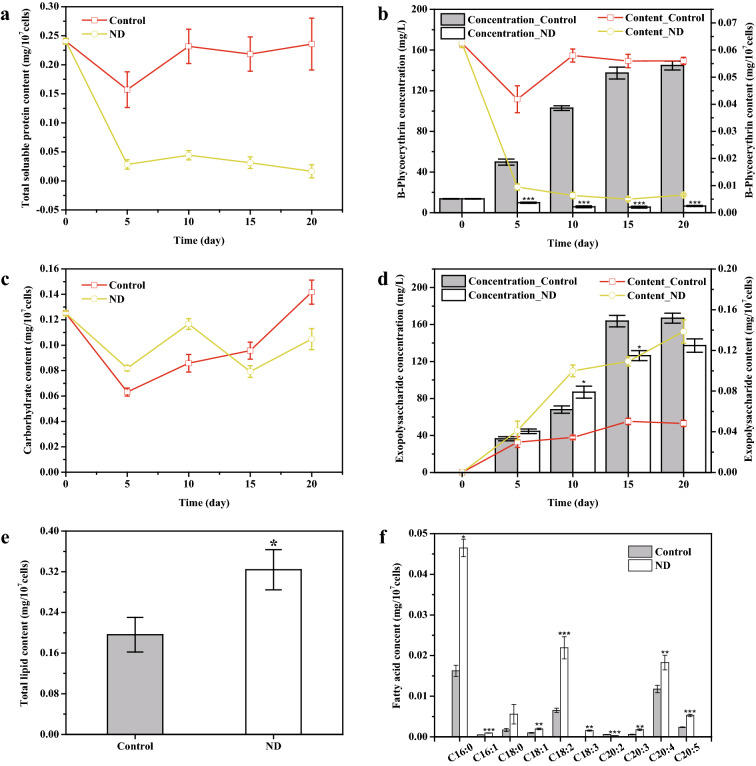
Changes of biochemical macromolecules and high-value active substances in *P. purpureum* under normal and nitrogen-deficient conditions. **a** Changes in total soluble protein (TSP) content. **b** Changes in B-phycoerythrin content and yield (concentration). **c** Changes in carbohydrate content. **d** Changes in exopolysaccharide content and yield (concentration). **e** Total lipid (TL) content of normal and the nitrogen-deficient cells at day 20. **f** Fatty acids (FAs) profiles of normal and the nitrogen-deficient cells at day 20. ND means nitrogen deprivation. Data are expressed as mean  ±  SD (*n*  =  3). *Significant difference (*t* test, *p*  <  0.05), **significant difference (*t *test, *p*  <  0.01), ***significant difference (*t *test, *p*  <  0.001)

In general, nitrogen-containing substances are recycled in the absence of nitrogen to produce essential proteins such as key enzymes (nitrogen transporters or nitrate reductase) or to provide molecular building blocks for cell survival, with an overall reduction in protein content (Corteggiani Carpinelli et al. 2014; Levitan et al. 2015). Under normal conditions, the intracellular TSP content of *P. purpureum* remained stable, but during the 5 days of nitrogen deficiency, the intracellular TSP content sharply dropped and finally reached equilibrium. On the 20th day, the intracellular TSP of the control group and the nitrogen deficiency group was 0.24  ±  0.04 mg/10^7^ cells and 0.02  ±  0.01 mg/10^7^ cells, respectively (Fig. 2a). Not surprisingly, nitrogen deficiency led to a decrease in the nitrogen-containing B-phycoerythrin and chlorophyll *a* content, which was most pronounced in the early stages of nitrogen deficiency (Fig. 2b; Additional file 1: Figure S2). Chlorophyll *a* is the key pigment in photosynthesis of green algae and higher plants, while phycobilisome plays a similar role in cyanobacteria and red algae (Adir et al. 2020; Ma et al., 2020), and phycoerythrin content in *P. purpureum* is much higher than chlorophyll *a* (Fig. 2b; Additional file 1: Figure S2), which may play a leading role in the capture and transfer of light energy. Reductions in B-phycoerythrin and chlorophyll *a* in *P. purpureum* caused by nitrogen deficiency may be the reason for the decrease of photosynthetic efficiency.

Despite the observed growth inhibition, large amounts of cell wall polysaccharides were synthesized by nitrogen-deficient cells of *P. purpureum*, which was considered a protective mechanism under stress (Arad et al. 1988). In this study, we observed that the carbohydrate content in both the control group and the nitrogen deficiency group decreased in the early stage (Fig. 2c), possibly because the cells were forced to secrete polysaccharides to adapt to the new environment (Fig. 2d). After the adaptation period (day 5), the carbohydrate content in the control group increased with growth, while that in the nitrogen-deficient group remained stable (Fig. 2c). Exopolysaccharide content increased under both conditions, but the accumulation rate was faster in the nitrogen-deficient group, reaching 0.14  ±  0.01 mg/10^7^ cells on day 20, which was 2.88 times that of the control group (Fig. 2d). Interestingly, the high yield of polysaccharides under normal conditions appears to be a combination of increased cell density and increased polysaccharide content in a single cell (Figs. 1a, 2d), which differs from B-phycoerythrin accumulation. Exopolysaccharide yield in the two groups was similar (166.90  ±  5.40 mg/L in the control group; 137.25  ±  7.19 mg/L in the nitrogen deficiency group), but the nitrogen deficiency group had fewer cells (Figs. 1a, 2d), which was more conducive to downstream separation and purification. In terms of total polysaccharides, *P. purpureum* tended to secrete more polysaccharides under nitrogen-deficient conditions.

Nitrogen stress can promote lipid accumulation, particularly TAG, which has been reported in many microalgae (Li et al. 2014; Liu et al. 2019). Compared with the control group, the total lipid (TL) content of *P. purpureum* in the nitrogen-deficient group increased by 65.2%, reaching as high as 0.32  ±  0.04 mg/10^7^ cells on the 20th day (Fig. 2e). The fatty acids with the highest content mainly included palmitic acid (PA), linolenic acid (LA), and ARA (Fig. 2f). Under nitrogen stress, the contents of total fatty acids (TFA) and individual fatty acids increased, except for eicosadienoic acid (C20:2), which decreased by 53% (Fig. 2f). Among these, the contents of PA and eicosatrienoic acid (C20:3) most significantly changed, which increased by 236% and 232%, respectively (Fig. 2f). We hypothesized that the increase in C20:3 might be related to the decrease in C20:2, as the substrate C18:2 shared by the two seems to flow more to C20:3 through the n-6 pathway. Although ARA content increased by 55%, EPA content increased by 123%, resulting in a decrease in the ARA to EPA ratio (ARA/EPA) from 4.97 to 3.50 (Fig. 2f; Table 1). Normally, PUFAs account for the highest proportion of total fatty acids in *P. purpureum*, while SFA and MUFAs accounted for the highest proportion in the nitrogen deficiency group (Table 1), indicating that although overall fatty acid content increased, unsaturation of total fatty acids decreased, which was concordant to the findings of previous reports (Msanne et al. 2012).

**Table 1:** Fatty acids composition of *P. purpureum* under normal and nitrogen deprivation conditions

	SFA (mg/10^9^ cells)	MUFA (mg/10^9^ cells)	PUFA (mg/10^9^ cells)	UFA (mg/10^9^ cells)	TFA (mg/10^9^ cells)	ARA/EPA
Control	1.79 ± 0.17	0.15 ± 0.01	2.19 ± 0.16	2.34 ± 0.17	4.13 ± 0.34	4.97 ± 0.27
ND	5.21 ± 0.18^a^	0.29 ± 0.03^c^	4.91 ± 0.52^c^	5.20 ± 0.55^c^	10.41 ± 1.73^b^	3.45 ± 0.14^b^

Data are expressed as mean  ±  SD (*n*  =  3)

*SFA* saturated fatty acid; *MUFA* monounsaturated fatty acid; *PUFA* polyunsaturated fatty acid; *UFA* unsaturated fatty acid; *TFA* total fatty acid; *ARA* arachidonic acid; *EPA* eicosapentaenoic acid

^a^Significant difference (*t *test, *p*  <  0.05)

^b^Significant difference (*t *test, *p*  <  0.01)

^c^Significant difference (*t* test, *p*  <  0.001)

### De novo transcriptome assembly and annotation and global gene expression analysis

To further elucidate the molecular mechanism by which *P. purpureum* responds to nitrogen deficiency, we performed time-resolved comparative transcriptome analysis, in which cells from normal and nitrogen deprivation conditions on days 5 and 15 were collected for RNA-seq analysis. Eight samples (two biological replicates per case) were used for transcriptome assembly, and a total of 80.06 Gb of clean data were obtained. The clean data of each sample reached 7.90 Gb, and the percentage of Q30 was at least 93.67%. The clean reads of each sample were mapped to the reference genome, with alignment efficiency ranging from 87 to 94% (Additional file 1: Table S1).

The annotation of gene function in each database was determined (Additional file 1: Figure S3; Additional file 2: Data S1). In terms of COG function classification, the most significant ones were “basic function prediction”, “ribosome regeneration”, and “signal transduction” (Additional file 1: Figure S4). In the “molecular function” category, the top four GO terms were “catalytic activity”, “binding”, “structural molecule activity”, and “transporter activity” (Additional file 1: Figure S5). For KEGG enrichment analysis, the number of genes related to “protein processing in the endoplasmic reticulum” ranked first (Additional file 1: Figure S6). In addition, we annotated 21 transcription factor (TF) families (183 genes), 18 transcription regulator (TR) families (94 genes), and 48 protein kinase (PK) families (202 genes). Among the predicted transcription factors, the number of C2H2 and MYB-related families was the highest, containing 66 and 32 genes, respectively. The GANT (23 genes) and SNF2 (17 genes) families were the predominant transcription regulatory factors. In terms of protein kinases, the CAMK-CDPK family (60 gens) was by far the most abundant (Additional file 3: Data S2). Studies have shown that the TF NIT2, which regulates nitrate metabolism, is upregulated sixfold under nitrogen-deficient conditions (Miller et al. 2010).

**Fig. 3:**
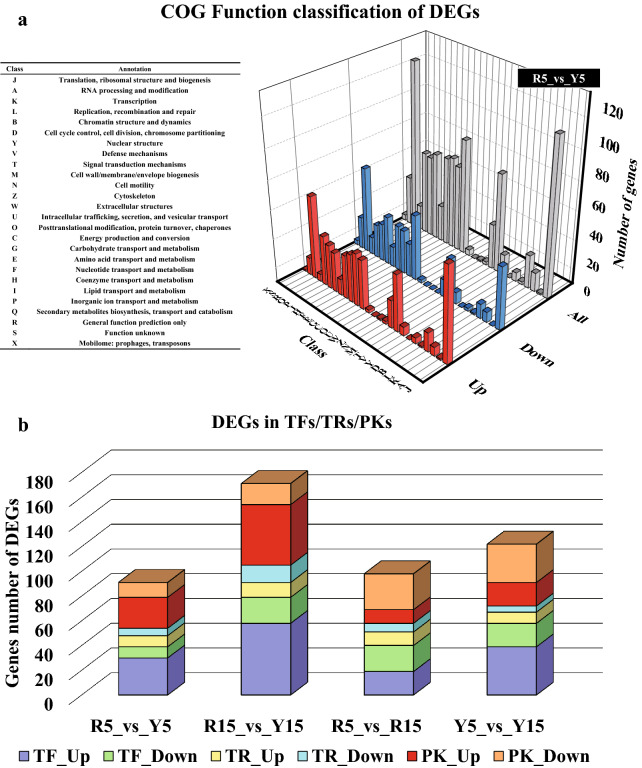
COG function classification and functional annotation of differentially expressed genes (DEGs). **a** COG function classification of DEGs on day 5. R5_vs_Y5 means gene set obtained by differential expression analysis on day 5, the upregulated (up) and downregulated (down) DEGs indicated that the expression level of the gene was significantly higher and lower (|Log_2_FC|> 1, FDR  < 0.05) on the 5th day of nitrogen deficiency (Y5) than that in the control group (R5), respectively. **b** Changes of the number of DEGs in TFs, TRs, and PKs. TFs means transcription factors; TRs means transcription regulators; PKs means protein kinases. TF_Up and TF_Down represent the upregulated and downregulated transcription factors, respectively, and so on

Differentially expressed genes (DEGs) are the main foci of transcriptome analysis. In this study, we adopted a comparative transcriptome approach to define the DEGs by comparing the gene expression between the nitrogen-deficient group and the control group at a specific time point (days 5 and 15). We further analyzed the enrichment of DEGs in the COG, GO, and KEGG pathways (Additional file 1: Figures S7–11), and the results showed that in the COG function classification, DEGs on day 5 (R5_vs_Y5) mainly enriched the categories of “general function prediction only”, “translation, ribosomal structure and biogenesis”, “posttranslational modification, protein turnover, chaperones”, and “signal transduction mechanisms” (Fig. 3a), which suggested the complex response mechanism of *P. purpureum* under the conditions of nitrogen deficiency. In addition, we analyzed gene expression levels of the annotated TRs, TFs, and PKs that have regulatory or signal transduction functions (Fig. 3b), and the highest number of DEGs was observed on the 15th day, indicating that *P. purpureum* had a more complex and urgent metabolic regulatory mechanism under nitrogen stress. Compared with normal conditions, most of the DEGs encoding TFs, TRs, and PKs were upregulated under nitrogen-deficient conditions (R5_vs_Y5, R15_vs_Y15), which was different from the expression of these genes during growth (R5_vs_R15, Y5_vs_Y15).

### Active chemical energy promotes metabolic migration

Consistent with previous observations in various microalgae (Miller et al. 2010; Sun et al. 2013), most photosynthesis-related proteins were significantly downregulated at prophase (day 5) under nitrogen deficiency. A total of 5 ATP synthase-encoding genes were annotated, all of which were downregulated on the 5th day, while two genes were upregulated on the 15th day, indicating the important role of these two genes in photosynthetic ATP production under nitrogen deficiency (Additional file 1: Table S2).

A total of 70 genes were annotated to oxidative phosphorylation, among which 30 genes were differentially expressed, including 25 upregulated genes and 5 downregulated genes (Additional file 1: Table S3). In addition, most of the ATP synthase-encoding genes were upregulated (Additional file 1: Table S3). The overall upregulation of oxidative phosphorylation- and ATP synthase-encoding genes indicates that the production of ATP under nitrogen-deficient conditions is mainly due to oxidative phosphorylation. It has been reported that oxidative phosphorylation is the main source of ATP under nitrogen stress (Schmollinger et al. 2014).

**Fig. 4:**
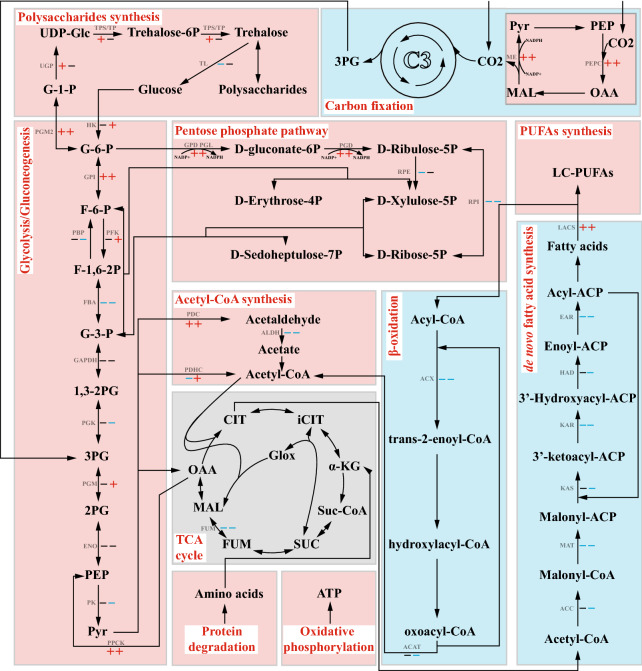
Regulation of genes involved in central carbon metabolism. This image shows central carbon metabolic pathways, including the Calvin cycle, gluconeogenesis, glycolysis, pentose phosphate pathway, and the tricarboxylic acid cycle. The red plus sign indicates upregulation, the blue minus sign indicates downregulation, and the black sign indicates no significant difference. The front and back positions indicate the differential expression at day 5 and day 15, respectively. The regions in red and blue indicate the upregulation and downregulation of the overall pathway, respectively. Abbreviations for metabolites were shown in black. PEP, phosphoenolpyruvate; OAA, oxaloacetate; MAL, malate; Pyr, pyruvate; 3PG, 3-phosphoglycerate; 1,3-2PG, 3-phospho-glycerol phosphate; G-3-P, glyceraldehyde 3-phosphate; F-1,6-2P, fructose 1,6-bisphosphate; F-6-P, fructose 6-phosphate; G-6-P, glucose 6-phosphate; G-1-P, glucose 1-phosphate; UDP-Glc, UDP-glucose; 2PG, 2-phospho-glycerate; CIT, citrate; iCIT, isocitrate; α-KG, 2-oxo-glutarate; Suc-CoA, succinyl-CoA; SUC, succinate; FUM, fumarate. Abbreviations for enzymes were shown in gray. PEPC, phosphoenolpyruvate carboxylase; ME, malic enzyme (malate dehydrogenase); PGK, phosphoglycerate kinase; GAPDH, glyceraldehyde 3-phosphate dehydrogenase; FBA, fructose-bisphosphate aldolase; PBP, fructose-1,6-bisphosphatase; GPI, glucose-6-phosphate isomerase; PGM2, phosphoglucomutase; UGP, UTP-glucose-1-phosphate uridylyltransferase; TPS/TP, trehalose 6-phosphate synthase/phosphatase; TL, α,α-trehalase; HK, hexokinase; PFK, phosphofructokinase; PGM, 2,3-diphosphoglycerate-independent phosphoglycerate mutase; ENO, enolase; PK, pyruvate kinase; PDHC, pyruvate dehydrogenase complex; PDC, pyruvate decarboxylase; ALDH, aldehyde dehydrogenase; FUM, fumarate hydratase; GPD, glucose-6-phosphate 1-dehydrogenase; PGL, 6-phosphogluconolactonase; PGD, 6-phosphogluconate dehydrogenase; RPE, ribulose-phosphate 3-epimerase; RPI, ribose-5-phosphate isomerase; ACC, acetyl-CoA carboxylase; MAT, malonyltransferase; KAS, β-ketoacyl-ACP synthase; KAR, β-ketoacyl-ACP reductase; HAD, β-hydroxyoctanoyl-ACP-dehydrase; EAR, enoyl-ACP reductase; LACS, long-chain acyl-CoA synthetase; ACX, acyl-CoA oxidase. ACAT, acetyl-CoA C-acetyltransferase

In the pentose phosphate pathway, GPD (glucose-6-phosphate 1-dehydrogenase) and PGL (6-phosphogluconolactonase) catalyze the conversion of G-6-P to gluconate-6P, which generates ribulose-5P under the catalysis of PGD (6-phosphogluconate dehydrogenase). These two steps are accompanied by the production of reducing power NADPH. The upregulation of the pentose phosphate pathway under nitrogen deficiency to provide reducing power for free fatty acid synthesis has been reported in microalgae (Jaeger et al. 2017; Liu et al. 2019). Two *GDP* genes and two *PGD* genes were annotated in *P. purpureum* that showed varying degrees of upregulation under nitrogen deprivation (Fig. 4; Additional file 1: Table S4). The expression of one *GPD* gene increased by nearly 89-fold (Log_2_FC  =  6.49), and another *PGD* gene was more than 500-fold upregulated (Log_2_FC  =  8.98), indicating the production of large amounts of NADPH in the pentose phosphate pathway (Fig. 4; Additional file 1: Table S4). In addition, not only the abovementioned ME (nearly four times upregulated) plays a role in carbon sequestration, but its catalytic process requires NADP  +  as the substrate to generate NADPH. The upregulation of *ME* provides reducing power for cell metabolism, which has long been reported in other microalgae (Jaeger et al. 2017).

### Carbon flow leads to the synthesis of polysaccharides and lipids in the prophase and anaphase, respectively

Among the 31 genes noted in the KEGG pathway for carbon fixation in photosynthetic organisms, 14 DEGs and 10 downregulated genes were found (Fig. 4; Additional file 1: Table S5). However, among the 14 DEGs, 4 genes were upregulated under nitrogen deficiency, including a glutamate glyoxylate aminotransferase-encoding gene *GGAT*, a phosphoenolpyruvate carboxylase-encoding gene *PEPC*, and two malic enzyme-encoding genes (*ME*) (Fig. 4; Additional file 1: Table S5). These are key genes in the C4 pathway, which are significantly upregulated in the process of nitrogen deficiency, suggesting that there may be a C4 pathway in *P. purpureum*, which plays a role in CO_2_ supplementation under nitrogen deprivation. Zhao et al. reported that four key genes in the C4 pathway were significantly upregulated (> 10-fold) under nitrogen-deficient conditions, suggesting that both C3 and C4 pathways exist in *Haematococcus pluvialis* (Zhao et al., 2020).

Phosphoenolpyruvate carboxykinase-encoding gene *PPCK* was significantly upregulated under nitrogen-deficient conditions on days 5 and 15, but the fructose-1,6-bisphosphatase-encoding gene *FBP*, another key enzyme in the gluconeogenic pathway, was significantly downregulated on day 15 (Log_2_FC  <  − 1.78), suggesting that gluconeogenesis is mainly upregulated at prophase (Fig. 4; Additional file 1: Table S6). In addition, during the conversion of hexose to disaccharide, we found that *UGP* (UTP-glucose-1-phosphate uridylyltransferase encoding gene) and *TPS/TP* (trehalose 6-phosphate synthase/phosphatase encoding genes) were upregulated on day 5 but downregulated on day 15 (Fig. 4; Additional file 1: Table S6). In response, the expression of the α,α-trehalase (*TL*) gene did not significantly change on day 15 from downregulation on day 5 (Fig. 4; Additional file 1: Table S6). The upregulation of gluconeogenesis and the downregulation of polysaccharide decomposition on day 5 indicate that polysaccharide synthesis is enhanced at the early stage, which was concordant to the observed increase in polysaccharide content.

There are three rate-limiting enzymes in glycolysis, namely, hexokinase (HK), 6-phospho-fructose-kinase (PFK), and pyruvate kinase (PK). Differential expression of two *HK* and *PFK* genes revealed opposite directions on the 15th day, but the upregulated extent was higher than the downregulated one (Fig. 4; Additional file 1: Table S6). Therefore, we believe that both *HK* and *PFK* were upregulated on the 15th day. However, one of the three genes encoding *PK* showed significant downregulation at day 15 (Fig. 4; Additional file 1: Table S6). In addition, the downregulation of *PGK* (phosphoglycerate kinase) on day 15, together with the upregulation of *PGM* (phosphoglycerate mutase) (Fig. 4; Additional file 1: Table S6), indicating that Calvin cycle-derived 3PG (3-phosphoglycerate) is directed towards glycolysis than gluconeogenesis, which has also been reported in *Monoraphidium neglectum* (Jaeger et al. 2017). Enhancement of polysaccharides catabolism and upregulation of glycolysis pathway genes indicate that glycolysis is accelerated at the late stage, resulting in the enhancement of pyruvate to acetyl-CoA synthesis.

The upregulation of the pyruvate dehydrogenase complex (PDHC) and PDHC bypass pathway under nitrogen-depleted conditions has been previously reported in the oleaginous microalga *Nannochloropsis oceanica* IMET1 (Li et al. 2014). Transcriptome analysis showed that although aldehyde dehydrogenase-encoding gene *ALDH* (Log2FC  =  − 1.23) was downregulated under nitrogen deprivation, pyruvate decarboxylase-encoding gene *PDC* (Log_2_FC  =  2.78) was significantly upregulated; hence, we believe that the PDHC bypass pathway is generally upregulated (Fig. 4; Additional file 1: Table S6). Furthermore, PDHC was downregulated on the 5th day and upregulated on the 15th day (Fig. 4; Additional file 1: Table S6). Therefore, we hypothesized that in the early stage of nitrogen deficiency, the generation of acetyl-CoA occurs mainly through the PDHC bypass pathway, and in the later stage, the PDHC and PDHC bypass pathways work together to accelerate the generation of acetyl-CoA and provide precursors for fatty acid synthesis.

There were no significant changes in most TCA cycle-related genes, except for two fumarate hydratase-encoding genes (*FUM*), suggesting that pyruvate-derived acetyl-CoA is directed towards free fatty acid synthesis than into the TCA cycle for complete oxidation (Fig. 4; Additional file 1: Table S7). Nevertheless, the carbon skeleton produced by protein degradation may enter the TCA cycle, increasing the TCA cycle from the substrate level and promoting oxidative phosphorylation (Additional file 4: Data S3). Most of the genes related to ubiquitin-mediated proteolysis were upregulated under nitrogen deprivation conditions, which indicated enhanced proteolysis (Additional file 1: Table S8). In addition, the genes related to aminoacyl-tRNA synthesis were downregulated, while the ribosomal protein-coding genes were upregulated (Additional file 1: Table S9). The downregulation of genes related to aminoacyl-tRNA synthesis indicates a decrease in aminoacyl-tRNAs; thus, more amino acids from protein degradation may enter the TCA cycle to provide a carbon skeleton for cell metabolism. We believe that ribosomal protein-encoding genes are upregulated because cells need to synthesize new proteins that play a key role in nitrogen stress such as nitrate reductase and nitrate transporter, which help cells absorb nitrogen sources from outside the cell (Additional file 1: Table S10).

Most genes involved in de novo synthesis of fatty acids were downregulated to varying degrees, including acetyl-CoA carboxylase-encoding *ACC*, malonyltransferase-encoding gene *MAT*, β -ketoacyl-ACp synthase-encoding gene *KAS*, β-ketoacyl-ACP reductase-encoding gene *KAR*, β-hydroxyoctanoyl-ACP-dehydrase-encoding gene *HAD*, and enoyl-ACP reductase-encoding gene *EAR* (Fig. 4; Additional file 1: Table S11). In terms of β-oxidation of fatty acids, acyl-CoA oxidase-encoding gene *ACX* exhibited the most significant downregulation (Fig. 4; Additional file 1: Table S11). In addition, enzymes directly involved in the degradation of carbon chains did not exhibit significant changes in expression, except for *ACAT*, the gene encoding acetyl-CoA C-acetyltransferase (Fig. 4; Additional file 1: Table S11).

Although the genes of de novo fatty acid synthesis pathway were downregulated, the increased content of acetyl-CoA, a precursor of lipid synthesis, derived from glycolysis pathway, and the downregulation of fatty acid β oxidation pathway could explain the lipid accumulation in *P. purpureum* during the late period of nitrogen deficiency. Besides, the downregulation of de novo fatty acid synthesis has also occurred in other microalgae, and the relatively plausible explanation is that gene expression in the de novo synthesis of fatty acids is normally excessive (Jaeger et al. 2017; Li et al. 2014; Liang et al. 2012,2019; Msanne et al. 2012).

### Proposed pathways for polysaccharide biosynthesis in *P. purpureum*

Unlike brown algae polysaccharides, which are composed of fucose or mannose (Ficko-Blean et al. 2015; Skriptsova 2015), the structure of *P. purpureum* polysaccharides is much more complex. Gloaguen et al. demonstrated by nuclear magnetic resonance (NMR) spectroscopy that the polysaccharides of *P. purpureum* are composed of three neutral monosaccharides (Xyl, Glc, and Gal) and one uronic acid (GlcA) (Gloaguen et al. 2004).

**Fig. 5:**
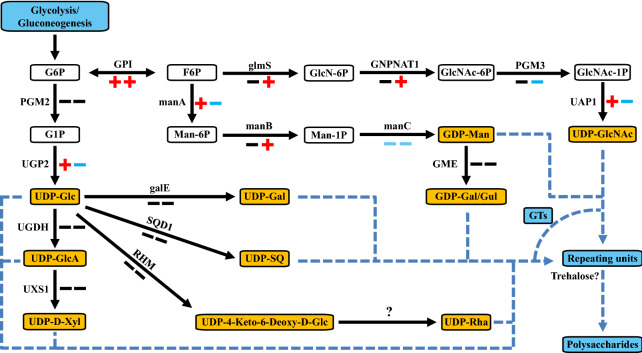
The interconversion between amino sugar and nucleotide sugar in *P. purpureum*. The red plus sign indicates upregulation, the blue minus sign indicates downregulation, and the black sign indicates no significant difference. The front and back positions indicate the differential expression at day 5 and day 15, respectively. G6P, glucose 6-phosphate; G1P, glucose 1-phosphate; UDP-Glc, UDP-glucose; UDP-GlcA, UDP-glucuronate; UDP-D-Xyl, UDP-D-xylose; UDP-Gal, UDP-galactose; UDP-SQ, UDP-sulfoquinovose; UDP-4-keto-deoxy-D-Glc, UDP-4-dehydro-6-deoxy-D-glucose; UDP-Rha, UDP-rhamnose; F6P, fructose 6-phosphate; GlcN-6P, glucosamine 6-phosphate; GlcNAc-6P, N-acetyl-D-glucosamine 6-phosphate; GlcNAc-1P, N-acetyl-D-glucosamine 1-phosphate; UDP-GlcNAc, UDP-N-acetyl-alpha-D-glucosamine; Man-6P, mannose 6-phosphate; Man-1P, mannose 1-phosphate; GDP-Man, GDP-mannose; GDP-Gal/Gul, GDP-galactose/gulose. PGM, phosphoglucomutase; UGP, UTP-glucose-1-phosphate uridylyltransferase; UGDH, UDP-glucose 6-dehydrogenase; UXS1, UDP-glucuronate decarboxylase; galE, UDP-glucose 4-epimerase; SQD1, UDP-sulfoquinovose synthase; RHM, UDP-glucose 4,6-dehydratase; GPI, glucose-6-phosphate isomerase; glmS, glutamine-fructose-6-phosphate transaminase (isomerizing); GNPNAT1, glucosamine-phosphate N-acetyltransferase; PGM3, phosphoacetylglucosamine mutase; UAP1, UDP-N-acetylglucosamine diphosphorylase; manA, mannose-6-phosphate isomerase; manB, phosphomannomutase; manC, mannose-1-phosphate guanylyltransferase; GME, GDP-D-mannose 3', 5'-epimerase. GTs, glycosyltransferases

To date, only a few studies on the synthesis pathway of polysaccharides from microalgae have been conducted. Here, from the perspective of amino and nucleotide sugars and carbohydrate-active enzymes (CAZymes), we mainly focused on the interconversion between glycosyl-donors and the differential expression of glycosyltransferase-encoding genes to elaborate the synthesis pathway of polysaccharides in *P. purpureum*. We believe that the synthesis of polysaccharides in *P. purpureum* begins with the synthesis of disaccharides (trehalose), and then the glycosyl donors are constantly connected to the repeated units with the catalysis of glycosyltransferase to form polysaccharides. According to the results of transcriptome annotation, there are nearly 10 different glycosyl donors in *P. purpureum* (Fig. 5). In addition to the previously reported monomer structure, there are also Man and GlcNAc (Fig. 5). We hypothesize that there may be other monosaccharides in *P. purpureum* polysaccharides, but the content is relatively low and undetectable. Among the 21 genes annotated by the transcriptome that related to glycosyl-donor interconversion, 12 genes were significantly differentially expressed under nitrogen deprivation conditions, and almost all these DEGs are closely related to the synthesis of GDP-Man and UDP-GlcNAc, suggesting that *P. purpureum* may regulate the composition and structure of polysaccharides to cope with nitrogen stress (Fig. 5).

As for the CAZymes, a total of 119 genes were annotated in the transcriptome, among which the number of genes encoding glycoside transferases (GTs) and glycoside hydrolases (GHs) was the largest (Table 2). This is consistent with the 83 GTs annotated in the genome information, which also indirectly reflects the complex polysaccharide structure in *P. purpureum* (Bhattacharya et al. 2013). For GHs, the number of significantly downregulated genes was higher than the number of upregulated genes, indicating that the decomposition of polysaccharides in *P. purpureum* was inhibited, which corresponds to the increase of its content (Table 2; Fig. 2d). As for the quantitative differences of carbohydrate-binding modules (CBMs) in the genome and transcriptome, we believe that some genes with inaccurate predictions at that time have been endowed with other functions, resulting in a decrease in the number of CBM-related genes (Table 2).

**Table 2:** CAZymes present in the *P. purpureum* transcriptome and genome

	GHs	GTs	PLs	CEs	CBMs	AAs
Transcriptome annotation	26 (0, 5, 0, 8)	72 (10, 3, 7, 14)	0 (0, 0, 0, 0)	4 (0, 0, 1, 0)	4 (0, 1, 0, 1)	11 (4, 3, 3, 3)
Genome assembly	31 (5, 5, 3, 8)	83 (8, 5, 4, 17)	0 (0, 0, 0, 0)	2 (0, 0, 1, 0)	40 (3, 4, 2, 4)	/

The first and third digits in brackets indicate the number of genes significantly upregulated on the 5th and 15th day of nitrogen deficiency, respectively. The second and fourth digits in brackets indicate the number of significantly downregulated differentially expressed genes on the 5th and 15th day of nitrogen deficiency, respectively

*GHs* glycoside hydrolases; *GTs* glycoside transferases; *PLs* polysaccharide lyases; *CEs* carbohydrate esterases; *CBM* carbohydrate-binding modules; *AAs* auxiliary activities

### Proposed pathways for ARA and EPA biosynthesis in *P. purpureum*

**Fig. 6:**
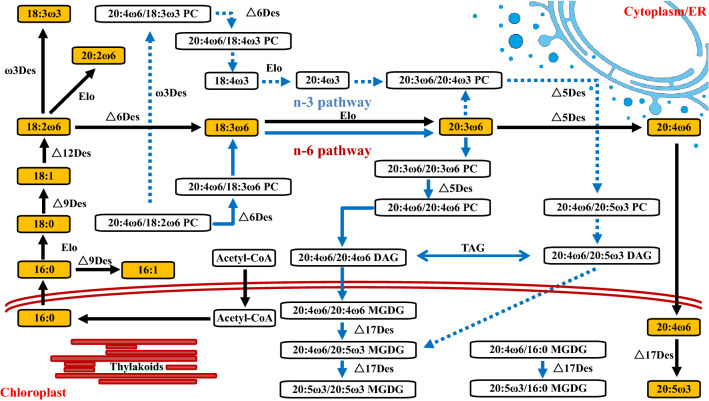
The proposed n-6 PUFAs synthesis pathway in *P. purpureum*. Dotted and solid lines in blue represent the n-3 and n-6 pathways reported in literature, respectively, while solid lines in black represent the PUFAs synthesis pathway proposed in this paper. The yellow solid boxes represent free fatty acids detected in this study, while the empty boxes represent lipids detected in the literature. Des, desaturase; Elo, elongase; PC, phosphatidylcholine; DAG, diacylglycerol; TAG, triacylglycerol; MGDG, monogalatosyl diglyceride; 16:0, C16:0; 16:1, C16:1(9); 18:0, C18:0; 18:1, C18:1(9); 18:2ω6, C18:2 (9,12); 18:3ω3, C18:2 (9,12,15); 18:3ω6, C18:3 (6,9,12); 18:4ω3, C18:4 (6,9,12,15); 20:2ω6, C20:2 (11,14); 20:3ω6, C20:3 (8,11,14); 20:4ω3, C20:4 (8,11,14,17); 20:4ω6, C20:4 (5,8,11,14); 20:5ω3, C20:5 (5,8,11,14,17)

As early as the late 1990s, researchers proposed two pathways for EPA synthesis in *P. purpureum*, namely, the n-3 and n-6 pathways (Khozin et al. 1997; Shiran et al. 1996). Recently, another possible Δ-8 pathway has been reported, suggesting that the intermediate metabolite of C18:2(9,12) and C20:3(8,11,14) is C20:2(11,14), i.e., the transformation from C18:2(9,12) to C20:3(8,11,14) is mediated by elongase and desaturase (Jiao et al. 2021). Based on the transcriptome annotation information and GC–MS analysis of the structure of free fatty acids, we are more inclined to believe that the synthesis of ARA and EPA in *P. purpureum* involves the n-6 synthesis pathway (Fig. 6; Table 3; Additional file 1: Figures S12–14).

**Table 3:** Profiles of gene expression related to PUFAs synthesis

Gene ID	Annotation	EC number	Log_2_FC^1^	FDR^1^	Log_2_FC^2^	FDR^2^
evm.TU.contig_2015.1	Delta-5 desaturase	[1.14.19.44]	1.2676	0.0000	0.4119	0.4522
evm.TU.contig_4413.10	Delta-5 desaturase	[1.14.19.44]	1.2263	0.0000	0.2928	0.5822
evm.TU.contig_2612.6	Delta-6 desaturase	[1.14.19.47]	2.5137	0.0000	1.8536	0.0007
evm.TU.contig_3456.6	Delta-9 desaturase	[1.14.19.1]	0.0856	0.8686	0.0797	0.8620
evm.TU.contig_2306.6	Delta-9 desaturase	[1.14.19.1]	2.6827	0.0000	0.4567	0.2159
evm.TU.contig_4413.10	Delta-9 desaturase	[1.14.19.1]	1.2263	0.0000	0.2928	0.5822
evm.TU.contig_3456.6	Delta-9 desaturase	[1.14.19.1]	0.0856	0.8686	0.0797	0.8620
evm.TU.contig_2077.16	Delta-12 desaturase	[1.14.19.22]	1.5136	0.0000	0.6626	0.3306
evm.TU.contig_2141.6	Omega-3/delta-17 desaturase	[1.14.19.25 1.14.19.35 1.14.19.36]	0.5550	0.1173	0.2708	0.7111
evm.TU.contig_3401.11	Elongase	[4.2.1.134]	0.4058	0.1514	0.8125	0.0242
evm.TU.contig_2016.10	Elongase	[4.2.1.134]	− 0.1256	0.7476	− 0.8224	0.0252
evm.TU.contig_491.2	Elongase	[1.1.1.330]	− 0.3860	0.5093	0.0708	0.8766
evm.TU.contig_3397.10	Elongase	[2.3.1.199]	0.7700	0.0096	0.4757	0.4303
evm.TU.contig_3572.1	Elongase	[2.3.1.199]	0.6492	0.0059	0.8123	0.0867
evm.TU.contig_4400.1	Elongase	[2.3.1.199 1.1.1.330 4.2.1.134 1.3.1.93]	0.0291	0.9247	− 0.4609	0.2637
evm.TU.contig_2039.9	Elongase	[2.3.1.199 1.1.1.330 4.2.1.134 1.3.1.93]	0.3041	0.4806	0.2574	0.4243

1 and 2 represent the differential expression on day 5 and day 15, respectively. Log_2_FC refers to the logarithm of the multiple of gene expression in the nitrogen-deficient group compared with that in the control group

The synthesis of PUFAs begins with the conversion of C16:0 to C16:1(9) and C18:0 to C18:1(9), both mediated by Δ9-Des, followed by the conversion of C18:1(9) to C18:2(9,12) as catalyzed by Δ12-Des (Fig. 6). These initial steps are consistent with most reports (Jiao et al. 2021; Khozin et al. 1997; Shiran et al. 1996). C18:2(9,12) can then be elongated by ELO to form C20:2(11,14) or desaturated by ω3-Des and Δ6-Des to form C18:3(9,12,15) and C18:3(6,9,12), respectively, but only C18:3(6,9,12) is directly related to the synthesis of ARA and EPA (Fig. 6). C18:3(6,9,12) can then generate C20:4(5,8,11,14) or ARA under the sequential action of ELO and Δ5-Des (Fig. 6). Finally, Δ17-Des (ω3-Des) further catalyzes the formation of EPA from ARA (Fig. 6). The above prediction is based on the types and structures of free fatty acids that were detected in this study. Because we did not detect an intermediate metabolite of the n-3 pathway, C18:4(6,9,12,15) (Khozin et al. 1997; Shiran et al. 1996), we believe that the n-3 pathway may not be present or may play a minor role in *P. purpureum*. Jiao et al. proposed the Δ-8 pathway based on the detection of EDA (C20:2), but the transcriptome did not annotate the ω8-Des-encoding gene that plays a key role in the Δ-8 pathway (Jiao et al. 2021). Taken together, we believe that the synthesis of ARA and EPA in *P. purpureum* is mainly through the n-6 pathway.

A total of 16 genes related to PUFA synthesis were annotated, including 9 desaturase-encoding genes and 7 elongase-encoding genes (Table 3). Most of the desaturase-encoding genes were significantly upregulated under nitrogen-deficient conditions, but the expression of elongase-encoding genes did not change (Table 3). The highest upregulation among all DEGs was one Δ9-Des-encoding gene (Log_2_FC > 2.6), which mediated the initiation of PUFAs synthesis; thus, it seemed that the de novo PUFAs synthesis was upregulated (Table 3). Two Δ5-Des-encoding genes were significantly upregulated under nitrogen deprivation (Log_2_FC  >  1.2) (Table 3), consistent with previous reports (Jiao et al. 2021). However, the upregulation of the Δ5-Des-encoding gene did not increase the ratio of ARA to EPA, which may be related to the substrate specificity of lipid synthesis (Liang et al. 2019), or it may be due to the degradation of EPA-rich membrane lipids caused by nitrogen stress, which indirectly increases the EPA pool.

## Conclusions

Under the condition of nitrogen deficiency, *P. purpureum* thickens the extracellular viscousness sheath by enhancing polysaccharide secretion, which is a self-protection mechanism. When subjected to nitrogen stress, *P. purpureum* immediately directed carbon flow to the synthesis of polysaccharides, and began to accumulate long-term storage substances lipids in the late stage of nitrogen deficiency. *P. purpureum* possesses a complex network of glycosyl-donor interconversion, which is consistent with its complex polysaccharide structure. In addition, the polyunsaturated fatty acid synthesis pathway of *P. purpureum* is more like the n-6 pathway. This is the first transcriptomic study of *P. purpureum*, which not only provides a production strategy for polysaccharides and fatty acids, but also provides targets for further genetic modification.

### Supplementary Information

**Additional file 1: Table S1.** Summary of reads in control and nitrogen deprivation of *P.* *purpureum* transcriptomes. **Table S2.** Profiles of gene expression related to photosynthesis. **Table S3.** Profiles of gene expression related to oxidative phosphorylation. **Table S4.** Profiles of gene expression related to pentose phosphate pathway. **Table S5.** Profiles of gene expression related to carbon fixation. **Table S6.** Profiles of gene expression related to gluconeogenesis and glycolysis. **Table S7.** Profiles of gene expression related to TCA cycle. **Table S8.** Profiles of gene expression related to ubiquitin mediated proteolysis. **Table S9.** Profiles of gene expression related to aminoacyl-tRNA biosynthesis. **Table S10.** Profiles of gene expression related to nitrogen metabolism. **Table S11.** Profiles of gene expression related to metabolism of fatty acids. **Figure S1.** Changes of Fv/Fm during growth. **Figure S2.** Changes of chlorophyll a content during growth. **Figure S3.** Transcriptome annotation in various databases. **Figure S4.** COG function classification of all genes. **Figure S5.** GO function classification of all genes. **Figure S6.** KEGG function classification of all genes. **Figure S7.** COG function classification of DEGs in R15_vs_Y15. **Figure S8.** GO function classification of DEGs in R5_vs_Y5. **Figure S9.** GO function classification of DEGs in R15_vs_Y15. **Figure S10.** KEGG function classification of DEGs in R5_vs_Y5. Figure S11. KEGG function classification of DEGs in R15_vs_Y15. **Figure S12.** GC-MS information of C18:3(9,12,15). **Figure S13.** GC-MS information of C18:3(6,9,12). **Figure S14.** GC-MS information of C20:3(11,14).

**Additional file 2: Data S1.** Expression profiles of all annotated genes.

**Additional file 3: Data S2.** Expression profiles of genes encoding TFs_TRs_PKs.

**Additional file 4: Data S3.** Expression profiles of genes encoding proteases and peptidases.

## Data Availability

The authors approved the availability of data and materials for publishing the manuscript. RNA-seq data have been deposited and are available under accession number PRJNA682303. Other data are provided as supplementary information.
