# Supplementary material for: Physiological and transcriptome analysis elucidates the metabolic mechanism of versatile Porphyridium purpureum under nitrogen deprivation for exopolysaccharides accumulation
Source: Bioresour Bioprocess. 2021 Aug 12;8(1):73. doi: 10.1186/s40643-021-00426-x (PMC10991915; doi:10.1186/s40643-021-00426-x)

**Supporting Information Description**

Table S1. Summary of reads in control and nitrogen deprivation of *P. purpureum* transcriptomes.

Table S2. Profiles of gene expression related to photosynthesis.

Table S3. Profiles of gene expression related to oxidative phosphotylation.

Table S4. Profiles of gene expression related to pentose phosphate pathway.

Table S5. Profiles of gene expression related to carbon fixation.

Table S6. Profiles of gene expression related to gluconeogenesis and glycolysis.

Table S7. Profiles of gene expression related to TCA cycle.

Table S8. Profiles of gene expression related to ubiquitin mediated proteolysis.

Table S9. Profiles of gene expression related to aminoacyl-tRNA biosynthesis.

Table S10. Profiles of gene expression related to nitrogen metabolism.

Table S11. Profiles of gene expression related to metabolism of fatty acids.

Figure S1. Changes of F_v_/F_m_ during growth.

Figure S2. Changes of chlorophyll *a* content during growth.

Figure S3. Transcriptome annotation in various databases.

Figure S4. COG function classification of all genes.

Figure S5. GO function classification of all genes.

Figure S6. KEGG function classification of all genes.

Figure S7. COG function classification of DEGs in R15_vs_Y15.

Figure S8. GO function classification of DEGs in R5_vs_Y5.

Figure S9. GO function classification of DEGs in R15_vs_Y15.

Figure S10. KEGG function classification of DEGs in R5_vs_Y5.

Figure S11. KEGG function classification of DEGs in R15_vs_Y15.

Figure S12. GC-MS information of C18:3(9,12,15).

Figure S13. GC-MS information of C18:3(6,9,12).

Figure S14. GC-MS information of C20:3(11,14).

Data S1. Expression profiles of all annotated genes.

Data S2. Expression profiles of genes encoding TFs_TRs_PKs.

Data S3. Expression profiles of genes encoding proteases and peptidases.

**Table S1. Summary of reads in control and nitrogen deprivation of *P. purpureum* transcriptomes.**

| Sample ID | ReadSum | BaseSum | GC (%) | N (%) | Q20 (%) | Q30 (%) | Total Reads | Mapped Reads | Ratio (%) |
| --- | --- | --- | --- | --- | --- | --- | --- | --- | --- |
| R15-1 | 38826335 | 11598250596 | 58.36 | 0 | 97.92 | 94.36 | 77652670 | 72433556 | 93.28 |
| R15-2 | 47600518 | 14209826782 | 58.16 | 0 | 97.93 | 94.33 | 95201036 | 89204592 | 93.70 |
| R5-1 | 29320985 | 8768012600 | 57.03 | 0 | 97.61 | 94.06 | 58641970 | 53270205 | 90.84 |
| R5-2 | 31120324 | 9316750642 | 54.69 | 0 | 97.38 | 93.67 | 62240648 | 55729731 | 89.54 |
| Y15-1 | 30696602 | 9172052192 | 57.54 | 0 | 98.29 | 95.17 | 61393204 | 54617311 | 88.96 |
| Y15-2 | 33860719 | 10130564654 | 57.65 | 0 | 98.18 | 94.91 | 67721438 | 59196668 | 87.41 |
| Y5-1 | 29966608 | 8964083494 | 57.05 | 0 | 97.8 | 94.22 | 59933216 | 54293134 | 90.59 |
| Y5-2 | 26397884 | 7900235410 | 57.24 | 0 | 98.07 | 94.73 | 52795768 | 47396312 | 89.77 |

R5 and R15 represent samples from day 5 and 15 under normal conditions, respectively. Y5 and Y15 represent samples from day 5 and 15 under nitrogen deprivation conditions, respectively.

**Table 2. Profiles of gene expression related photosynthesis.**

| Gene ID | Annotation | Log_2_FC^1^ | FDR^1^ | Log_2_FC^2^ | FDR^2^ |
| --- | --- | --- | --- | --- | --- |
| Porphyridium_purpureum_newGene_1689 | photosystem II 13kDa protein, Psb28 | -4.766 | 0.0000 | -- | -- |
| evm.TU.contig_3695.1 | photosystem I subunit PsaO | -3.929 | 0.0000 | -1.4270 | 0.0241 |
| evm.TU.contig_3802.1 | photosystem II PsbM protein | -3.550 | 0.0000 | -1.6447 | 0.0035 |
| evm.TU.contig_2142.1 | light-harvesting complex I chlorophyll *a*/*b* binding protein 1 | -3.428 | 0.0001 | -1.7756 | 0.0234 |
| evm.TU.contig_4542.1 | photosystem II Psb27 protein | -3.384 | 0.0000 | -1.6947 | 0.0000 |
| evm.TU.contig_2493.3 | light-harvesting complex I chlorophyll *a*/*b* binding protein 1 | -3.308 | 0.0000 | -2.4672 | 0.0001 |
| evm.TU.contig_4487.13 | photosystem II oxygen-evolving enhancer protein 2, PsbP | -2.527 | 0.0000 | -0.1238 | 0.7639 |
| evm.TU.contig_469.1 | photosystem II oxygen-evolving enhancer protein 3, PsbQ | -2.495 | 0.0000 | 0.3368 | 0.6740 |
| evm.TU.contig_3421.1 | light-harvesting complex I chlorophyll *a*/*b* binding protein 1 | -2.188 | 0.0001 | 0.1377 | 0.8154 |
| evm.TU.contig_2297.1 | photosystem II CP47 chlorophyll apoprotein, PsbB | -2.146 | 0.0338 | 0.4108 | 0.7263 |
| evm.TU.contig_435.19 | light-harvesting complex I chlorophyll *a*/*b* binding protein 1 | -1.885 | 0.0001 | -1.6714 | 0.0032 |
| evm.TU.contig_4406.7 | light-harvesting complex I chlorophyll *a*/*b* binding protein 1 | -1.872 | 0.0000 | -0.7019 | 0.0966 |
| evm.TU.contig_556.1 | photosystem II P680 reaction center D1 protein, PsbA | -1.842 | 0.0763 | -1.8731 | 0.0007 |
| evm.TU.contig_2663.4 | photosystem II oxygen-evolving enhancer protein 1, PsbO | -1.700 | 0.0001 | -0.6868 | 0.0360 |
| evm.TU.contig_555.1 | photosystem II PsbU protein | -1.532 | 0.0000 | 0.1118 | 0.8699 |
| evm.TU.contig_2113.4 | phycobilisome core linker protein, ApcC | -1.416 | 0.0000 | -0.3003 | 0.5699 |
| evm.TU.contig_2124.1 | F-type H^+^-transporting ATPase subunit alpha | -1.296 | 0.2502 | 1.3899 | 0.0816 |
| evm.TU.contig_776.1 | light-harvesting complex I chlorophyll *a*/*b* binding protein 1 | -1.007 | 0.0090 | 0.7287 | 0.1155 |
| evm.TU.contig_2092.11 | F-type H^+^-transporting ATPase subunit gamma | -0.930 | 0.0001 | 0.9047 | 0.0425 |
| evm.TU.contig_2146.6 | phycocyanobilin lyase subunit beta, CpcF | -0.924 | 0.0088 | -1.2716 | 0.0121 |
| evm.TU.contig_2186.5 | ferredoxin-NADP^+^ reductase, PetH | -0.750 | 0.2311 | -0.5671 | 0.2253 |

**Table 2. Profiles of gene expression related photosynthesis (continued).**

| Gene ID | Annotation | Log_2_FC^1^ | FDR^1^ | Log_2_FC^2^ | FDR^2^ |
| --- | --- | --- | --- | --- | --- |
| Porphyridium_purpureum_newGene_1643 | F-type H^+^-transporting ATPase subunit beta | -0.724 | 0.5667 | 2.8020 | 0.0006 |
| evm.TU.contig_2104.20 | F-type H^+^-transporting ATPase subunit gamma | -0.275 | 0.5529 | -1.3133 | 0.0012 |
| Porphyridium_purpureum_newGene_1622 | F-type H^+^-transporting ATPase subunit beta | -0.268 | 0.8294 | -- | -- |
| evm.TU.contig_491.7 | light-harvesting complex I chlorophyll *a*/*b* binding protein 1 | -0.174 | 0.8960 | 0.0851 | 0.9594 |
| evm.TU.contig_487.4 | cytochrome c6, PetJ | 0.835 | 0.4966 | -0.9499 | 0.5318 |
| evm.TU.contig_529.1 | cytochrome b6-f complex iron-sulfur subunit, PetC | 0.980 | 0.0001 | 2.4555 | 0.0000 |
| evm.TU.contig_490.2 | phycocyanobilin lyase subunit alpha, CpcE | 1.972 | 0.0000 | 0.7911 | 0.1021 |
| evm.TU.contig_592.2 | ferredoxin, PetF | 6.101 | 0.0000 | -1.7556 | 0.0000 |

^1^ and ^2^ represent the differential expression on day 5 and day 15, respectively. Log_2_FC refers to the logarithm of the multiple of gene expression in the nitrogen-deficient group compared with that in the control group.

**Table S3. Profiles of gene expression related to oxidative phosphotylation.**

| Gene ID | Annotation | FDR^1^ | Log_2_FC^1^ | FDR^2^ | Log_2_FC^2^ |
| --- | --- | --- | --- | --- | --- |
| evm.TU.contig_2019.6 | V-type H^+^-transporting ATPase subunit C | 0.5137 | 0.3506 | 0.0682 | 0.7789 |
| evm.TU.contig_2130.13 | succinate dehydrogenase (ubiquinone) flavoprotein subunit | 0.3366 | -0.3704 | 0.1156 | -0.4692 |
| evm.TU.contig_569.1 | succinate dehydrogenase (ubiquinone) iron-sulfur subunit | 0.2325 | 0.5157 | 0.0284 | -0.9111 |
| evm.TU.contig_560.6 | ubiquinol-cytochrome c reductase iron-sulfur subunit | 0.0000 | 1.2101 | 0.0000 | 1.6726 |
| Porphyridium_purpureum_newGene_109 | ubiquinol-cytochrome c reductase cytochrome b subunit | 0.0203 | -2.4536 | 0.6346 | 0.6393 |
| evm.TU.contig_3957.1 | ubiquinol-cytochrome c reductase cytochrome c1 subunit | 0.0000 | 1.3702 | 0.0000 | 2.8302 |
| evm.TU.contig_4410.24 | ubiquinol-cytochrome c reductase subunit 7 | 0.0000 | 1.0336 | 0.0000 | 2.0851 |
| evm.TU.contig_2139.24 | ubiquinol-cytochrome c reductase subunit 8 | 0.0122 | 0.6319 | 0.0004 | 1.6972 |
| evm.TU.contig_3511.1 | ubiquinol-cytochrome c reductase subunit 9 | 0.0000 | 1.1480 | 0.0000 | 1.8905 |
| evm.TU.contig_3427.10 | polyphosphate kinase | 0.2283 | -0.3327 | 0.0000 | -1.9097 |
| evm.TU.contig_3416.19 | inorganic pyrophosphatase | 0.9132 | -0.0488 | 0.0000 | -2.0645 |
| evm.TU.contig_4507.1 | inorganic pyrophosphatase | 0.0207 | 0.6938 | 0.0000 | 3.3603 |
| evm.TU.contig_3409.10 | H^+^-transporting ATPase | 0.0000 | 1.8242 | 0.3912 | 1.3142 |
| evm.TU.contig_4410.18 | H^+^-transporting ATPase | 0.0000 | 1.4837 | 0.0000 | 3.6824 |
| evm.TU.contig_2124.1 | H^+^-transporting ATPase subunit alpha | 0.2502 | -1.2964 | 0.0816 | 1.3899 |
| Porphyridium_purpureum_newGene_1622 | H^+^-transporting ATPase subunit beta | 0.8294 | -0.2684 | -- | -- |
| Porphyridium_purpureum_newGene_1643 | H^+^-transporting ATPase subunit beta | 0.5667 | -0.7242 | 0.0006 | 2.8020 |
| evm.TU.contig_2104.20 | H^+^-transporting ATPase subunit gamma | 0.5529 | -0.2748 | 0.0012 | -1.3133 |

**Table S3.** **Profiles of gene expression related to oxidative phosphotylation (continued).**

| Gene ID | Annotation | FDR^1^ | Log_2_FC^1^ | FDR^2^ | Log_2_FC^2^ |
| --- | --- | --- | --- | --- | --- |
| evm.TU.contig_2092.11 | H^+^-transporting ATPase subunit gamma | 0.0001 | -0.9297 | 0.0425 | 0.9047 |
| evm.TU.contig_732.3 | F-type H^+^-transporting ATPase subunit alpha | 0.7622 | 0.1777 | 0.5017 | 0.3147 |
| evm.TU.contig_3410.3 | F-type H^+^-transporting ATPase subunit beta | 0.6107 | 0.2554 | 0.1161 | -0.5940 |
| evm.TU.contig_2068.20 | F-type H^+^-transporting ATPase subunit delta | 0.0673 | -0.4832 | 0.5360 | 0.2850 |
| evm.TU.contig_456.1 | F-type H^+^-transporting ATPase subunit gamma | 0.4332 | -0.3381 | 0.8707 | -0.0870 |
| evm.TU.contig_3654.2 | F-type H^+^-transporting ATPase subunit O | 0.2074 | 0.3688 | 0.1959 | 0.5616 |
| evm.TU.contig_4463.3 | V-type H^+^-transporting ATPase subunit H | 0.2512 | 0.4989 | 0.5749 | -0.2208 |
| evm.TU.contig_3748.1 | V-type H^+^-transporting ATPase subunit A | 0.0000 | 1.2282 | 0.2544 | -0.3154 |
| evm.TU.contig_3489.2 | V-type H^+^-transporting ATPase subunit A | 0.9629 | 0.0219 | 0.0069 | 1.1843 |
| evm.TU.contig_2305.6 | V-type H^+^-transporting ATPase subunit d | 0.0787 | 0.7382 | 0.1945 | 0.6012 |
| evm.TU.contig_4434.6 | V-type H^+^-transporting ATPase subunit B | 0.0391 | 0.9805 | 0.0372 | 0.9906 |
| evm.TU.contig_4499.2 | V-type H^+^-transporting ATPase subunit D | 0.0066 | 0.6659 | 0.1739 | 0.4976 |
| evm.TU.contig_2059.36 | V-type H^+^-transporting ATPase subunit E | 0.7480 | 0.1264 | 0.1742 | -0.6046 |
| evm.TU.contig_623.2 | V-type H^+^-transporting ATPase subunit F | 0.0092 | 0.5998 | 0.0000 | 1.9636 |
| evm.TU.contig_4463.2 | V-type H^+^-transporting ATPase subunit G | 0.0115 | -0.6627 | 0.6084 | 0.2913 |
| evm.TU.contig_565.6 | V-type H^+^-transporting ATPase subunit a | 0.3587 | 0.3857 | 0.4496 | 0.2910 |
| evm.TU.contig_2713.1 | V-type H^+^-transporting ATPase subunit a | 0.3123 | 0.3242 | 0.8585 | 0.0735 |
| evm.TU.contig_2059.26 | V-type H^+^-transporting ATPase subunit a | 0.3205 | 0.3154 | 0.6547 | -0.1694 |

**Table S3. Profiles of gene expression related to oxidative phosphotylation (continued).**

| Gene ID | Annotation | FDR^1^ | Log_2_FC^1^ | FDR^2^ | Log_2_FC^2^ |
| --- | --- | --- | --- | --- | --- |
| evm.TU.contig_2025.71 | V-type H^+^-transporting ATPase subunit a | 0.7837 | 0.3508 | 0.8695 | -0.0791 |
| evm.TU.contig_2185.8 | V-type H^+^-transporting ATPase 16kDa proteolipid subunit | 0.9147 | -0.0383 | 0.0000 | 1.6110 |
| Porphyridium_purpureum_newGene_120 | cytochrome c oxidase subunit 1 | 0.4675 | -0.8895 | 0.1827 | 1.5654 |
| evm.TU.contig_4422.1 | protoheme IX farnesyltransferase | 0.0139 | 0.5590 | 0.7983 | -0.1204 |
| evm.TU.contig_2146.8 | cytochrome c oxidase assembly protein subunit 11 | 0.8071 | -0.0805 | 0.0002 | -1.3365 |
| evm.TU.contig_4448.4 | cytochrome c oxidase subunit 5b | 0.5137 | 0.3506 | 0.0682 | 0.7789 |
| evm.TU.contig_2110.5 | cytochrome c oxidase subunit 6a | 0.3366 | -0.3704 | 0.1156 | -0.4692 |
| evm.TU.contig_4432.13 | V-type H^+^-transporting ATPase 21kDa proteolipid subunit | 0.2325 | 0.5157 | 0.0284 | -0.9111 |
| Porphyridium_purpureum_newGene_381 | NADH-ubiquinone oxidoreductase chain 1 | 0.0000 | 1.2101 | 0.0000 | 1.6726 |
| Porphyridium_purpureum_newGene_37 | NADH-ubiquinone oxidoreductase chain 2 | 0.0203 | -2.4536 | 0.6346 | 0.6393 |
| evm.TU.contig_3384.4 | NADH dehydrogenase | 0.0000 | 1.3702 | 0.0000 | 2.8302 |
| evm.TU.contig_2070.25 | NADH dehydrogenase (ubiquinone) Fe-S protein 1 | 0.0000 | 1.0336 | 0.0000 | 2.0851 |
| evm.TU.contig_494.4 | NADH dehydrogenase (ubiquinone) Fe-S protein 1 | 0.0122 | 0.6319 | 0.0004 | 1.6972 |
| evm.TU.contig_442.6 | NADH dehydrogenase (ubiquinone) Fe-S protein 2 | 0.0000 | 1.1480 | 0.0000 | 1.8905 |
| evm.TU.contig_2048.5 | NADH dehydrogenase (ubiquinone) Fe-S protein 3 | 0.2283 | -0.3327 | 0.0000 | -1.9097 |
| evm.TU.contig_2068.13 | NADH dehydrogenase (ubiquinone) Fe-S protein 4 | 0.9132 | -0.0488 | 0.0000 | -2.0645 |
| evm.TU.contig_502.6 | NADH dehydrogenase (ubiquinone) Fe-S protein 5 | 0.0207 | 0.6938 | 0.0000 | 3.3603 |
| evm.TU.contig_2102.15 | NADH dehydrogenase (ubiquinone) Fe-S protein 6 | 0.0000 | 1.8242 | 0.3912 | 1.3142 |

**Table S3. Profiles of gene expression related to oxidative phosphotylation (continued).**

| Gene ID | Annotation | FDR^1^ | Log_2_FC^1^ | FDR^2^ | Log_2_FC^2^ |
| --- | --- | --- | --- | --- | --- |
| evm.TU.contig_510.12 | NADH dehydrogenase (ubiquinone) Fe-S protein 7 | 0.0000 | 1.4837 | 0.0000 | 3.6824 |
| evm.TU.contig_2362.1 | NADH dehydrogenase (ubiquinone) Fe-S protein 8 | 0.2502 | -1.2964 | 0.0816 | 1.3899 |
| evm.TU.contig_719.1 | NADH dehydrogenase (ubiquinone) flavoprotein 1 | 0.8294 | -0.2684 | -- | -- |
| evm.TU.contig_3442.9 | NADH dehydrogenase (ubiquinone) flavoprotein 1 | 0.5667 | -0.7242 | 0.0006 | 2.8020 |
| evm.TU.contig_4432.16 | NADH dehydrogenase (ubiquinone) flavoprotein 2 | 0.5529 | -0.2748 | 0.0012 | -1.3133 |
| evm.TU.contig_3569.2 | NADH dehydrogenase (ubiquinone) 1 alpha subcomplex subunit 1 | 0.0001 | -0.9297 | 0.0425 | 0.9047 |
| evm.TU.contig_2288.7 | NADH dehydrogenase (ubiquinone) 1 alpha subcomplex subunit 2 | 0.7622 | 0.1777 | 0.5017 | 0.3147 |
| evm.TU.contig_452.12 | NADH dehydrogenase (ubiquinone) 1 alpha subcomplex subunit 5 | 0.6107 | 0.2554 | 0.1161 | -0.5940 |
| evm.TU.contig_2024.3 | NADH dehydrogenase (ubiquinone) 1 alpha subcomplex subunit 6 | 0.0673 | -0.4832 | 0.5360 | 0.2850 |
| evm.TU.contig_2094.15 | NADH dehydrogenase (ubiquinone) 1 alpha subcomplex subunit 8 | 0.4332 | -0.3381 | 0.8707 | -0.0870 |
| evm.TU.contig_2109.3 | NADH dehydrogenase (ubiquinone) 1 alpha subcomplex subunit 9 | 0.2074 | 0.3688 | 0.1959 | 0.5616 |
| evm.TU.contig_4398.7 | NADH dehydrogenase (ubiquinone) 1 alpha/beta subcomplex 1 | 0.2512 | 0.4989 | 0.5749 | -0.2208 |
| evm.TU.contig_4400.8 | NADH dehydrogenase (ubiquinone) 1 alpha/beta subcomplex 1 | 0.0000 | 1.2282 | 0.2544 | -0.3154 |
| evm.TU.contig_597.1 | NADH dehydrogenase (ubiquinone) 1 beta subcomplex subunit 9 | 0.9629 | 0.0219 | 0.0069 | 1.1843 |
| evm.TU.contig_532.8 | NADH dehydrogenase (ubiquinone) 1 alpha subcomplex subunit 12 | 0.0787 | 0.7382 | 0.1945 | 0.6012 |
| evm.TU.contig_2069.5 | NADH dehydrogenase (ubiquinone) 1 alpha subcomplex subunit 13 | 0.0391 | 0.9805 | 0.0372 | 0.9906 |

^1^ and ^2^ represent the differential expression on day 5 and day 15, respectively. Log_2_FC refers to the logarithm of the multiple of gene expression in the nitrogen-deficient group compared with that in the normal group.

**Table S4.** **Profiles of gene expression related to pentose phosphate pathway.**

| Gene ID | Annotation | FDR^1^ | Log_2_FC^1^ | FDR^2^ | Log_2_FC^2^ |
| --- | --- | --- | --- | --- | --- |
| evm.TU.contig_2118.8 | glucose-6-phosphate 1-dehydrogenase | 0.0000 | 1.8103 | 0.4516 | 0.2703 |
| evm.TU.contig_2186.3 | glucose-6-phosphate 1-dehydrogenase | 0.0000 | 1.7351 | 0.0000 | 6.4858 |
| evm.TU.contig_4419.10 | 6-phosphogluconolactonase | 0.1655 | -0.4371 | 0.5266 | -0.3669 |
| evm.TU.contig_4437.9 | 6-phosphogluconate dehydrogenase | 0.0096 | 1.0306 | 0.6952 | 0.1657 |
| evm.TU.contig_4463.1 | 6-phosphogluconate dehydrogenase | 0.2705 | 0.3661 | 0.0000 | 8.9800 |
| evm.TU.contig_2015.15 | ribulose-phosphate 3-epimerase | 0.0001 | 0.7875 | 0.0175 | 0.6379 |
| evm.TU.contig_617.2 | ribulose-phosphate 3-epimerase | 0.0000 | -1.4497 | 0.3756 | -0.3827 |
| evm.TU.contig_592.4 | ribose 5-phosphate isomerase A | 0.0000 | -2.0399 | 0.0024 | -0.8460 |
| evm.TU.contig_4449.1 | transketolase | 0.9387 | 0.0470 | 0.2936 | -0.5733 |
| evm.TU.contig_484.1 | fructose-bisphosphate aldolase, class I | 0.0000 | -2.1293 | 0.0000 | -2.7660 |
| evm.TU.contig_3490.7 | fructose-bisphosphate aldolase, class I | 0.3649 | -0.5623 | 0.9616 | 0.0248 |
| evm.TU.contig_3452.4 | fructose-bisphosphate aldolase, class II | 0.7141 | -0.2180 | 0.0109 | -1.1562 |
| evm.TU.contig_3540.3 | fructose-bisphosphate aldolase, class I | 0.0000 | -1.5100 | 0.1308 | 0.5699 |
| evm.TU.contig_2082.11 | diphosphate-dependent phosphofructokinase | 0.0000 | -3.0135 | 0.9891 | -0.0257 |
| evm.TU.contig_3490.10 | fructose-1,6-bisphosphatase I | 0.0015 | -0.7157 | 0.1220 | 0.6493 |
| evm.TU.contig_3540.6 | fructose-1,6-bisphosphatase I | 0.0008 | -0.7750 | 0.7983 | -0.1191 |
| evm.TU.contig_3402.5 | fructose-1,6-bisphosphatase I | 0.5630 | -0.3036 | 0.0020 | -1.7865 |
| evm.TU.contig_479.13 | 6-phosphofructokinase 1 | 0.0007 | 0.8324 | 0.0000 | 2.1775 |

**Table S4. Profiles of gene expression related to pentose phosphate pathway (continued).**

| Gene ID | Annotation | FDR^1^ | Log_2_FC^1^ | FDR^2^ | Log_2_FC^2^ |
| --- | --- | --- | --- | --- | --- |
| evm.TU.contig_2010.1 | 6-phosphofructokinase 1 | 0.0673 | 0.4279 | 0.0015 | -1.1796 |
| evm.TU.contig_3437.2 | glucose-6-phosphate isomerase | 0.0000 | 1.6848 | 0.0000 | 7.3794 |
| evm.TU.contig_2062.10 | glucose-6-phosphate isomerase | 0.6876 | -0.2252 | 0.1510 | 0.5791 |
| evm.TU.contig_2062.15 | ribose-phosphate pyrophosphokinase | 0.0100 | 1.3377 | 0.0005 | 2.8632 |
| evm.TU.contig_3490.2 | ribose-phosphate pyrophosphokinase | 0.0003 | -0.7729 | 0.0763 | -0.5881 |
| evm.TU.contig_452.2 | phosphoglucomutase | 0.1935 | -0.4642 | 0.4922 | -0.2768 |
| evm.TU.contig_3397.5 | phosphoglucomutase | 0.0204 | 0.7679 | 0.1136 | -0.9221 |
| evm.TU.contig_2051.32 | 2-dehydro-3-deoxyphosphogluconate aldolase/(4S)-4-hydroxy-2-oxoglutarate aldolase | 0.0001 | 1.7451 | 0.0073 | 1.7847 |
| evm.TU.contig_2065.6 | glyceraldehyde-3-phosphate dehydrogenase (NADP^+^) | 0.4419 | -0.3773 | 0.0000 | -2.5362 |

^1^ and ^2^ represent the differential expression on day 5 and day 15, respectively. Log_2_FC refers to the logarithm of the multiple of gene expression in the nitrogen-deficient group compared with that in the normal group.

**Table S5. Profiles of gene expression related to carbon fixation.**

| Gene ID | Annotation | FDR^1^ | Log_2_FC^1^ | FDR^2^ | Log_2_FC^2^ |
| --- | --- | --- | --- | --- | --- |
| evm.TU.contig_2015.15 | ribulose-phosphate 3-epimerase | 0.0001 | 0.7875 | 0.0175 | 0.6379 |
| evm.TU.contig_548.5 | aspartate aminotransferase, mitochondrial | 0.0051 | -0.7285 | 0.8484 | 0.0763 |
| evm.TU.contig_4445.8 | glutamate-glyoxylate aminotransferase | 0.0628 | 1.5396 | 0.0033 | 3.8692 |
| evm.TU.contig_617.2 | ribulose-phosphate 3-epimerase | 0.0000 | -1.4497 | 0.3756 | -0.3827 |
| evm.TU.contig_536.1 | malate dehydrogenase (oxaloacetate-decarboxylating) (NADP^+^) | 0.0000 | 2.4433 | 0.0003 | 1.9959 |
| evm.TU.contig_3540.6 | fructose-1,6-bisphosphatase I | 0.0008 | -0.7750 | 0.7983 | -0.1191 |
| evm.TU.contig_3540.3 | fructose-bisphosphate aldolase, class I | 0.0000 | -1.5100 | 0.1308 | 0.5699 |
| evm.TU.contig_3598.1 | phosphoribulokinase | 0.1360 | -0.7936 | 0.2034 | -0.7132 |
| evm.TU.contig_2306.3 | triosephosphate isomerase (TIM) | 0.0000 | -1.7962 | 0.0000 | -1.8983 |
| evm.TU.contig_462.12 | triosephosphate isomerase (TIM) | 0.0000 | -2.0766 | 0.0000 | -5.2682 |
| evm.TU.contig_3402.5 | fructose-1,6-bisphosphatase I | 0.5630 | -0.3036 | 0.0020 | -1.7865 |
| evm.TU.contig_2305.8 | malate dehydrogenase | 0.2821 | -0.5665 | 0.4390 | 0.4811 |
| evm.TU.contig_513.2 | alanine transaminase | 0.9127 | 0.1568 | 0.8646 | -0.2923 |
| evm.TU.contig_2044.16 | phosphoglycerate kinase | 0.2450 | 0.5891 | 0.5653 | 0.3133 |
| evm.TU.contig_3567.1 | phosphoglycerate kinase | 0.0626 | -0.8413 | 0.0071 | -1.5760 |
| evm.TU.contig_484.1 | fructose-bisphosphate aldolase, class I | 0.0000 | -2.1293 | 0.0000 | -2.7660 |
| evm.TU.contig_2505.4 | malate dehydrogenase | 0.8646 | -0.0787 | 0.8542 | -0.0854 |
| evm.TU.contig_3490.7 | fructose-bisphosphate aldolase, class I | 0.3649 | -0.5623 | 0.9616 | 0.0248 |
| evm.TU.contig_2354.2 | phosphoenolpyruvate carboxylase | 0.0001 | 1.4438 | 0.0000 | 1.7447 |
| evm.TU.contig_3490.10 | fructose-1,6-bisphosphatase I | 0.0015 | -0.7157 | 0.1220 | 0.6493 |
| evm.TU.contig_2393.1 | glyceraldehyde 3-phosphate dehydrogenase | 0.3542 | -0.6205 | 0.7753 | 0.1516 |

**Table S5. Profiles of gene expression related to carbon fixation (continued).**

| Gene ID | Annotation | FDR^1^ | Log_2_FC^1^ | FDR^2^ | Log_2_FC^2^ |
| --- | --- | --- | --- | --- | --- |
| evm.TU.contig_3690.1 | sedoheptulose-bisphosphatase | 0.5195 | -0.3378 | 0.6593 | -0.2128 |
| evm.TU.contig_743.1 | malate dehydrogenase (oxaloacetate-decarboxylating) (NADP^+^) | 0.0087 | 0.8001 | 0.0000 | 1.2691 |
| evm.TU.contig_487.3 | ribulose-bisphosphate carboxylase large chain | 0.2059 | -1.4320 | 0.1229 | 1.5666 |
| evm.TU.contig_774.2 | aspartate aminotransferase, cytoplasmic | 0.5182 | -0.2821 | 0.5166 | 0.2479 |
| evm.TU.contig_448.20 | malate dehydrogenase | 0.3571 | -0.4148 | 0.1411 | -0.5975 |
| evm.TU.contig_4449.1 | transketolase | 0.9387 | 0.0470 | 0.2936 | -0.5733 |
| evm.TU.contig_3585.3 | sedoheptulose-bisphosphatase | 0.2342 | -0.3482 | 0.0133 | -0.8389 |
| evm.TU.contig_592.4 | ribose 5-phosphate isomerase A | 0.0000 | -2.0399 | 0.0024 | -0.8460 |
| evm.TU.contig_548.3 | phosphoglycerate kinase | 0.0530 | -0.8207 | 0.0093 | -1.0270 |
| evm.TU.contig_3452.4 | fructose-bisphosphate aldolase, class II | 0.7141 | -0.2180 | 0.0109 | -1.1562 |
| evm.TU.contig_2015.15 | ribulose-phosphate 3-epimerase | 0.0001 | 0.7875 | 0.0175 | 0.6379 |
| evm.TU.contig_548.5 | aspartate aminotransferase, mitochondrial | 0.0051 | -0.7285 | 0.8484 | 0.0763 |
| evm.TU.contig_4445.8 | glutamate--glyoxylate aminotransferase | 0.0628 | 1.5396 | 0.0033 | 3.8692 |
| evm.TU.contig_617.2 | ribulose-phosphate 3-epimerase | 0.0000 | -1.4497 | 0.3756 | -0.3827 |
| evm.TU.contig_536.1 | malate dehydrogenase (oxaloacetate-decarboxylating) (NADP^+^) | 0.0000 | 2.4433 | 0.0003 | 1.9959 |
| evm.TU.contig_3540.6 | fructose-1,6-bisphosphatase I | 0.0008 | -0.7750 | 0.7983 | -0.1191 |
| evm.TU.contig_3540.3 | fructose-bisphosphate aldolase, class I | 0.0000 | -1.5100 | 0.1308 | 0.5699 |
| evm.TU.contig_3598.1 | phosphoribulokinase | 0.1360 | -0.7936 | 0.2034 | -0.7132 |

^1^ and ^2^ represent the differential expression on day 5 and day 15, respectively. Log_2_FC refers to the logarithm of the multiple of gene expression in the nitrogen-deficient group compared with that in the normal group.

**Table S6. Profiles of gene expression related to gluconeogenesis and glycolysis.**

| Gene ID | Annotation | FDR^1^ | Log_2_FC^1^ | FDR^2^ | Log_2_FC^2^ |
| --- | --- | --- | --- | --- | --- |
| evm.TU.contig_471.1 | pyruvate carboxylase | 0.0032 | -0.7543 | 0.0215 | -0.8059 |
| evm.TU.contig_3399.6 | phosphoenolpyruvate carboxykinase | 0.0004 | 1.3363 | 0.0008 | 1.9367 |
| evm.TU.contig_2075.7 | glucokinase | 0.3545 | 0.3783 | 0.0007 | -1.2612 |
| evm.TU.contig_564.6 | glucokinase | 0.9492 | -0.0533 | 0.0004 | 2.0686 |
| evm.TU.contig_3437.2 | glucose-6-phosphate isomerase | 0.0000 | 1.6848 | 0.0000 | 7.3794 |
| evm.TU.contig_2062.10 | glucose-6-phosphate isomerase | 0.6876 | -0.2252 | 0.1510 | 0.5791 |
| evm.TU.contig_2082.11 | diphosphate-dependent phosphofructokinase | 0.0000 | -3.0135 | 0.9891 | -0.0257 |
| evm.TU.contig_479.13 | 6-phosphofructokinase 1 | 0.0007 | 0.8324 | 0.0000 | 2.1775 |
| evm.TU.contig_2010.1 | 6-phosphofructokinase 1 | 0.0673 | 0.4279 | 0.0015 | -1.1796 |
| evm.TU.contig_484.1 | fructose-bisphosphate aldolase, class I | 0.0000 | -2.1293 | 0.0000 | -2.7660 |
| evm.TU.contig_3490.7 | fructose-bisphosphate aldolase, class I | 0.3649 | -0.5623 | 0.9616 | 0.0248 |
| evm.TU.contig_3452.4 | fructose-bisphosphate aldolase, class II | 0.7141 | -0.2180 | 0.0109 | -1.1562 |
| evm.TU.contig_3540.3 | fructose-bisphosphate aldolase, class I | 0.0000 | -1.5100 | 0.1308 | 0.5699 |
| evm.TU.contig_2393.1 | glyceraldehyde 3-phosphate dehydrogenase | 0.3542 | -0.6205 | 0.7753 | 0.1516 |
| evm.TU.contig_2044.16 | phosphoglycerate kinase | 0.2450 | 0.5891 | 0.5653 | 0.3133 |
| evm.TU.contig_3567.1 | phosphoglycerate kinase | 0.0626 | -0.8413 | 0.0071 | -1.5760 |
| evm.TU.contig_548.3 | phosphoglycerate kinase | 0.0530 | -0.8207 | 0.0093 | -1.0270 |
| evm.TU.contig_2065.6 | glyceraldehyde-3-phosphate dehydrogenase (NADP^+^) | 0.4419 | -0.3773 | 0.0000 | -2.5362 |

**Table S6.** **Profiles of gene expression related to gluconeogenesis and glycolysis (continued).**

| Gene ID | Annotation | FDR^1^ | Log_2_FC^1^ | FDR^2^ | Log_2_FC^2^ |
| --- | --- | --- | --- | --- | --- |
| evm.TU.contig_787.2 | probable phosphoglycerate mutase | 0.8957 | 0.0446 | 0.0003 | 1.0655 |
| evm.TU.contig_3491.9 | probable phosphoglycerate mutase | 0.0001 | -1.0594 | 0.3709 | -0.4032 |
| evm.TU.contig_549.2 | 2,3-bisphosphoglycerate-independent phosphoglycerate mutase | 0.3369 | 0.5097 | 0.0671 | -0.7662 |
| evm.TU.contig_2098.13 | pyruvate kinase | 0.6195 | -0.2579 | 0.0000 | -1.1181 |
| evm.TU.contig_2307.1 | pyruvate kinase | 0.3190 | 0.4528 | 0.4179 | 0.3334 |
| evm.TU.contig_3426.24 | pyruvate kinase | 0.1381 | 0.7301 | 0.0299 | 0.6229 |
| evm.TU.contig_441.25 | pyruvate dehydrogenase E1 component beta subunit | 0.0104 | -0.7361 | 0.6748 | -0.1474 |
| evm.TU.contig_2069.3 | pyruvate dehydrogenase E1 component alpha subunit | 0.4185 | -0.4155 | 0.3053 | -0.4134 |
| evm.TU.contig_3590.2 | pyruvate dehydrogenase E2 component (dihydrolipoamide acetyltransferase) | 0.4434 | -0.4410 | 0.0334 | -0.8970 |
| evm.TU.contig_4408.20 | pyruvate dehydrogenase E2 component (dihydrolipoamide acetyltransferase) | 0.1408 | -0.5428 | 0.0211 | -0.8391 |
| evm.TU.contig_3401.29 | pyruvate dehydrogenase E2 component (dihydrolipoamide acetyltransferase) | 0.3429 | -0.3572 | 0.0000 | 2.2740 |
| evm.TU.contig_4455.4 | pyruvate dehydrogenase E2 component (dihydrolipoamide acetyltransferase) | 0.0000 | -1.4153 | 0.0001 | -1.1189 |
| evm.TU.contig_2142.8 | dihydrolipoamide dehydrogenase | 0.2787 | -0.4595 | 0.4375 | -0.2203 |
| evm.TU.contig_2268.4 | dihydrolipoamide dehydrogenase | 0.0000 | -1.7357 | 0.0625 | -1.0308 |
| evm.TU.contig_2095.7 | dihydrolipoamide dehydrogenase | 0.0547 | -2.0723 | 0.0000 | -6.1884 |
| evm.TU.contig_479.1 | pyruvate decarboxylase | 0.0000 | 3.2041 | 0.0000 | 2.3621 |
| evm.TU.contig_2292.7 | aldehyde dehydrogenase (NAD^+^) | 0.6930 | 0.1639 | 0.9535 | -0.0251 |
| evm.TU.contig_563.3 | aldehyde dehydrogenase (NAD^+^) | 0.9968 | -0.0061 | 0.9394 | 0.1361 |

**Table S6. Profiles of gene expression related to gluconeogenesis and glycolysis(continued).**

| Gene ID | Annotation | FDR^1^ | Log_2_FC^1^ | FDR^2^ | Log_2_FC^2^ |
| --- | --- | --- | --- | --- | --- |
| evm.TU.contig_563.2 | aldehyde dehydrogenase (NAD^+^) | 0.6416 | -0.6000 | 0.6164 | -0.6595 |
| evm.TU.contig_456.3 | aldehyde dehydrogenase family 7 member A1 | 0.0026 | -1.1714 | 0.0000 | -1.2835 |
| evm.TU.contig_3407.4 | acetyl-CoA synthetase | 0.1686 | -0.3022 | 0.5264 | -0.2240 |
| evm.TU.contig_4516.3 | S-(hydroxymethyl)glutathione dehydrogenase/alcohol dehydrogenase | 0.0176 | -1.0571 | 0.0000 | -2.6577 |
| evm.TU.contig_3402.5 | fructose-1,6-bisphosphatase I | 0.5630 | -0.3036 | 0.0020 | -1.7865 |
| evm.TU.contig_2104.17 | aldose 1-epimerase | 0.6627 | 0.3981 | 0.9733 | 0.0346 |
| evm.TU.contig_452.2 | phosphoglucomutase | 0.1935 | -0.4642 | 0.4922 | -0.2768 |
| evm.TU.contig_3490.10 | fructose-1,6-bisphosphatase I | 0.0015 | -0.7157 | 0.1220 | 0.6493 |
| evm.TU.contig_2092.2 | enolase | 0.3527 | -0.5836 | 0.6344 | -0.2721 |
| evm.TU.contig_2057.14 | enolase | 0.8260 | -0.1253 | 0.7923 | 0.1458 |
| evm.TU.contig_634.1 | glucose-6-phosphate 1-epimerase | 0.0000 | -3.0917 | 0.0396 | -1.5613 |
| evm.TU.contig_479.5 | glucose-6-phosphate 1-epimerase | 0.4931 | -0.2111 | 0.8781 | -0.0539 |
| evm.TU.contig_2029.6 | glucose-6-phosphate 1-epimerase | 0.0000 | -2.2169 | 0.0031 | -0.9896 |

^1^ and ^2^ represent the differential expression on day 5 and day 15, respectively. Log_2_FC refers to the logarithm of the multiple of gene expression in the nitrogen-deficient group compared with that in the normal group.

**Table S7. Profiles of gene expression related to TCA cycle.**

| Gene ID | Annotation | FDR^1^ | Log_2_FC^1^ | FDR^2^ | Log_2_FC^2^ |
| --- | --- | --- | --- | --- | --- |
| evm.TU.contig_441.25 | pyruvate dehydrogenase E1 component beta subunit | 0.0104 | -0.7361 | 0.6748 | -0.1474 |
| evm.TU.contig_2069.3 | pyruvate dehydrogenase E1 component alpha subunit | 0.4185 | -0.4155 | 0.3053 | -0.4134 |
| evm.TU.contig_3590.2 | pyruvate dehydrogenase E2 component | 0.4434 | -0.4410 | 0.0334 | -0.8970 |
| evm.TU.contig_4408.20 | pyruvate dehydrogenase E2 component | 0.1408 | -0.5428 | 0.0211 | -0.8391 |
| evm.TU.contig_3401.29 | pyruvate dehydrogenase E2 component | 0.3429 | -0.3572 | 0.0000 | 2.2740 |
| evm.TU.contig_4455.4 | pyruvate dehydrogenase E2 component | 0.0000 | -1.4153 | 0.0001 | -1.1189 |
| evm.TU.contig_2142.8 | dihydrolipoamide dehydrogenase | 0.2787 | -0.4595 | 0.4375 | -0.2203 |
| evm.TU.contig_2268.4 | dihydrolipoamide dehydrogenase | 0.0000 | -1.7357 | 0.0625 | -1.0308 |
| evm.TU.contig_2095.7 | dihydrolipoamide dehydrogenase | 0.0547 | -2.0723 | 0.0000 | -6.1884 |
| evm.TU.contig_471.1 | pyruvate carboxylase | 0.0032 | -0.7543 | 0.0215 | -0.8059 |
| evm.TU.contig_2161.2 | citrate synthase | 0.9321 | -0.0274 | 0.0278 | -0.6778 |
| evm.TU.contig_4483.7 | citrate synthase | 0.5581 | 0.6420 | 0.1176 | -2.2436 |
| evm.TU.contig_4438.23 | citrate synthase | 0.0004 | -1.0309 | 0.0657 | -0.6883 |
| evm.TU.contig_4422.6 | citrate synthase | 0.0174 | -0.5704 | 0.6991 | 0.2509 |
| evm.TU.contig_2032.4 | ATP citrate (pro-S)-lyase | 0.0061 | 0.8404 | 0.1853 | -0.7940 |
| evm.TU.contig_2032.23 | ATP citrate (pro-S)-lyase | 0.2818 | 0.5013 | 0.9911 | -0.0081 |
| evm.TU.contig_3426.11 | aconitate hydratase | 0.0201 | 0.8805 | 0.2808 | -0.6083 |
| evm.TU.contig_4488.4 | aconitate hydratase | 0.3531 | -0.3984 | 0.0089 | -0.9219 |

**Table S7. Profiles of gene expression related to TCA cycle (continued).**

| Gene ID | Annotation | FDR^1^ | Log_2_FC^1^ | FDR^2^ | Log_2_FC^2^ |
| --- | --- | --- | --- | --- | --- |
| evm.TU.contig_4400.7 | isocitrate dehydrogenase (NAD^+^) | 0.8141 | 0.1550 | 0.6410 | -0.2328 |
| evm.TU.contig_2090.27 | isocitrate dehydrogenase (NAD^+^) | 0.9101 | 0.0858 | 0.8341 | -0.1351 |
| evm.TU.contig_2105.1 | 2-oxoglutarate dehydrogenase E1 component | 0.0059 | -0.8017 | 0.2548 | -0.5239 |
| evm.TU.contig_573.1 | 2-oxoglutarate dehydrogenase E2 component | 0.4527 | -0.3180 | 0.1514 | 0.3969 |
| evm.TU.contig_2189.4 | succinyl-CoA synthetase alpha subunit | 0.2159 | -0.4972 | 0.2811 | -0.6655 |
| evm.TU.contig_2107.1 | succinyl-CoA synthetase beta subunit | 0.0162 | -0.7915 | 0.0762 | -0.7902 |
| evm.TU.contig_569.1 | succinate dehydrogenase (ubiquinone) iron-sulfur subunit | 0.2325 | 0.5157 | 0.0284 | -0.9111 |
| evm.TU.contig_2130.13 | succinate dehydrogenase (ubiquinone) flavoprotein subunit | 0.3366 | -0.3704 | 0.1156 | -0.4692 |
| Porphyridium_purpureum_newGene_1035 | fumarate hydratase, class II | 0.0000 | -2.3878 | 0.2320 | -1.7229 |
| evm.TU.contig_3450.6 | fumarate hydratase, class I | 0.0013 | -1.0650 | 0.0000 | -1.8025 |
| evm.TU.contig_2505.4 | malate dehydrogenase | 0.8646 | -0.0787 | 0.8542 | -0.0854 |
| evm.TU.contig_448.20 | malate dehydrogenase | 0.3571 | -0.4148 | 0.1411 | -0.5975 |
| evm.TU.contig_2305.8 | malate dehydrogenase | 0.2821 | -0.5665 | 0.4390 | 0.4811 |
| evm.TU.contig_2189.4 | succinyl-CoA synthetase alpha subunit | 0.2159 | -0.4972 | 0.2811 | -0.6655 |

^1^ and ^2^ represent the differential expression on day 5 and day 15, respectively. Log_2_FC refers to the logarithm of the multiple of gene expression in the nitrogen-deficient group compared with that in the normal group.

**Table S8. Profiles of gene expression related to ubiquitin mediated proteolysis.**

| Gene ID | Annotation | FDR^1^ | Log_2_FC^1^ | FDR^2^ | Log_2_FC^2^ |
| --- | --- | --- | --- | --- | --- |
| evm.TU.contig_494.6 | S-phase kinase-associated protein 1 | 0.0006 | 1.2212 | 0.0041 | 0.8019 |
| evm.TU.contig_494.7 | S-phase kinase-associated protein 1 | 0.9206 | -0.0344 | 0.0074 | 0.9032 |
| evm.TU.contig_2295.1 | ubiquitin-activating enzyme E1 | 0.7971 | 0.0900 | 0.4439 | 0.3012 |
| evm.TU.contig_865.5 | cullin 1 | 0.1079 | 0.5058 | 0.2024 | -0.4869 |
| evm.TU.contig_2038.1 | cullin 1 | 0.4005 | -0.3443 | 0.0017 | -1.5177 |
| evm.TU.contig_2106.4 | anaphase-promoting complex subunit 1 | 0.0573 | 0.5595 | 0.1956 | 0.4630 |
| evm.TU.contig_2070.15 | anaphase-promoting complex subunit 2 | 0.0045 | -0.6700 | 0.1628 | -0.5372 |
| evm.TU.contig_498.2 | anaphase-promoting complex subunit 3 | 0.1045 | -0.5643 | 0.0042 | -1.0183 |
| evm.TU.contig_3468.15 | anaphase-promoting complex subunit 6 | 0.0184 | -0.6787 | 0.4664 | -0.2620 |
| evm.TU.contig_2030.18 | anaphase-promoting complex subunit 8 | 0.2504 | -0.4483 | 0.1883 | -0.5466 |
| evm.TU.contig_4398.2 | anaphase-promoting complex subunit 10 | 0.4326 | 0.3252 | 0.0783 | -0.5901 |
| evm.TU.contig_2294.2 | cell division cycle 20, cofactor of APC complex | 0.8447 | 0.1047 | 0.0964 | 0.7243 |
| evm.TU.contig_503.4 | cell division cycle 20-like protein 1, cofactor of APC complex | 0.8930 | -0.0397 | 0.9248 | -0.0359 |
| evm.TU.contig_4416.16 | cullin 3 | 0.4492 | 0.1736 | 0.2572 | -0.3523 |
| evm.TU.contig_2384.1 | transcription elongation factor B, polypeptide 1 | 0.3865 | 0.3381 | 0.0455 | -0.7846 |
| evm.TU.contig_3466.2 | ubiquitin-conjugating enzyme E2 J2 | 0.1996 | -0.3176 | 0.2017 | -0.3681 |
| evm.TU.contig_2055.5 | E3 SUMO-protein ligase PIAS1 | 0.1020 | 0.5447 | 0.0217 | -0.9992 |
| evm.TU.contig_4545.1 | ubiquitin-conjugating enzyme E2 C | 0.8671 | 0.0979 | 0.9577 | 0.0492 |

**Table S8. Profiles of gene expression related to ubiquitin mediated proteolysis (continued).**

| Gene ID | Annotation | FDR^1^ | Log_2_FC^1^ | FDR^2^ | Log_2_FC^2^ |
| --- | --- | --- | --- | --- | --- |
| evm.TU.contig_2280.2 | ubiquitin-conjugating enzyme E2 D | 0.0013 | 0.9089 | 0.0000 | 2.7392 |
| evm.TU.contig_3435.19 | E3 ubiquitin-protein ligase RFWD2 | 0.0049 | 0.8372 | 0.0318 | -0.9649 |
| evm.TU.contig_516.7 | DNA excision repair protein ERCC-8 | 0.4545 | -0.2996 | 0.3775 | -0.4393 |
| evm.TU.contig_3391.7 | de-etiolated-1 | 0.0039 | 0.9476 | 0.0000 | 1.5851 |
| evm.TU.contig_2041.3 | ubiquitin-conjugating enzyme E2 A | 0.0292 | 0.6873 | 0.2827 | 0.4515 |
| evm.TU.contig_598.5 | ubiquitin-conjugating enzyme E2 A | 0.0004 | 0.8805 | 0.0146 | 1.0641 |
| Porphyridium_purpureum_newGene_1432 | ubiquitin-conjugating enzyme E2 A | 0.4100 | -0.5476 | 0.0073 | 1.7490 |
| Porphyridium_purpureum_newGene_1422 | ubiquitin-conjugating enzyme E2 A | 0.0847 | 0.7168 | 0.0000 | 1.5014 |
| evm.TU.contig_448.25 | ubiquitin-conjugating enzyme E2 G1 | 0.4913 | -0.1841 | 0.0005 | 1.1669 |
| evm.TU.contig_569.3 | ubiquitin-conjugating enzyme E2 H | 0.8235 | 0.0619 | 0.0000 | 1.4547 |
| evm.TU.contig_2255.3 | ubiquitin-conjugating enzyme E2 I | 0.3047 | -0.4837 | 0.9477 | -0.0400 |
| evm.TU.contig_3510.7 | ubiquitin-conjugating enzyme E2 J1 | 0.0902 | 0.5574 | 0.0000 | 1.7759 |
| evm.TU.contig_3531.2 | ubiquitin-conjugating enzyme E2 M | 0.0001 | 1.1622 | 0.0447 | 0.7654 |
| evm.TU.contig_2239.1 | ubiquitin-conjugating enzyme E2 N | 0.7992 | -0.3364 | 0.3668 | 1.3776 |
| evm.TU.contig_4527.2 | ubiquitin-conjugating enzyme E2 O | -- | -- | -- | -- |
| evm.TU.contig_3512.3 | ubiquitin-conjugating enzyme E2 S | 0.4170 | 0.4291 | 0.0000 | 1.3084 |
| evm.TU.contig_657.1 | ubiquitin-protein ligase E3 C | 0.0093 | 0.9895 | 0.9917 | -0.0048 |
| evm.TU.contig_623.3 | E3 ubiquitin-protein ligase TRIP12 | 0.0009 | 0.7807 | 0.0681 | 0.7822 |

**Table S8. Profiles of gene expression related to ubiquitin mediated proteolysis (continued).**

| Gene ID | Annotation | FDR^1^ | Log_2_FC^1^ | FDR^2^ | Log_2_FC^2^ |
| --- | --- | --- | --- | --- | --- |
| evm.TU.contig_2051.21 | E3 ubiquitin-protein ligase HUWE1 | 0.9618 | 0.0178 | 0.0046 | -1.0536 |
| evm.TU.contig_3384.13 | ubiquitin conjugation factor E4 B | 0.0186 | 0.5896 | 0.3935 | -0.2672 |
| evm.TU.contig_2510.2 | pre-mRNA-processing factor 19 | 0.0283 | -0.4921 | 0.6702 | 0.1541 |
| evm.TU.contig_479.7 | E3 ubiquitin-protein ligase synoviolin | 0.8902 | 0.0509 | 0.0000 | 1.5768 |
| evm.TU.contig_2039.6 | E3 ubiquitin-protein ligase FANCL | 0.0325 | 0.7371 | 0.0021 | -1.1628 |
| evm.TU.contig_519.6 | cullin 4 | 0.8689 | -0.0531 | 0.0001 | -1.0361 |
| evm.TU.contig_3484.2 | DNA damage-binding protein 1 | 0.1935 | -0.2884 | 0.0017 | -0.9056 |
| evm.TU.contig_3452.3 | E3 ubiquitin-protein ligase HERC4 | 0.9993 | -0.0004 | 0.8335 | -0.0777 |
| evm.TU.contig_2024.26 | ubiquitin-like 1-activating enzyme E1 A | 0.2027 | 0.4745 | 0.8044 | -0.1229 |
| evm.TU.contig_2051.15 | ubiquitin-like 1-activating enzyme E1 B | 0.0001 | -1.0134 | 0.0389 | -0.8049 |
| evm.TU.contig_580.1 | ubiquitin-activating enzyme E1 C | 0.0112 | 0.5055 | 0.0009 | 1.0192 |
| evm.TU.contig_456.9 | ubiquitin-conjugating enzyme E2 W | 0.0002 | 0.8538 | 0.0004 | 1.0344 |
| evm.TU.contig_2272.4 | ubiquitin-conjugating enzyme E2 E | 0.3335 | 0.3760 | 0.7884 | -0.1179 |

^1^ and ^2^ represent the differential expression on day 5 and day 15, respectively. Log_2_FC refers to the logarithm of the multiple of gene expression in the nitrogen-deficient group compared with that in the normal group.

**Table S9. Profiles of gene expression related to aminoacyl-tRNA biosynthesis.**

| Gene ID | Annotation | FDR^1^ | Log_2_FC^1^ | FDR^2^ | Log_2_FC^2^ |
| --- | --- | --- | --- | --- | --- |
| evm.TU.contig_583.6 | threonyl-tRNA synthetase | 0.6345 | 0.2663 | 0.2036 | 0.2807 |
| evm.TU.contig_2025.9 | alanyl-tRNA synthetase | 0.0386 | -1.1683 | 0.0057 | -0.7984 |
| evm.TU.contig_2284.17 | tyrosyl-tRNA synthetase | 0.1045 | 0.9160 | 0.5172 | 0.1973 |
| evm.TU.contig_444.25 | O-phospho-L-seryl-tRNASec:L-selenocysteinyl-tRNA synthase | 0.9954 | -0.0063 | 0.0008 | -1.2249 |
| evm.TU.contig_3414.3 | lysyl-tRNA synthetase, class II | 0.8362 | 0.0956 | 0.0001 | 0.8584 |
| evm.TU.contig_2173.14 | aspartyl-tRNA synthetase | 0.9351 | 0.1451 | 0.1518 | -1.5328 |
| evm.TU.contig_2194.8 | cysteinyl-tRNA synthetase | 0.2640 | 1.2322 | 0.4694 | 0.7539 |
| evm.TU.contig_453.1 | methionyl-tRNA formyltransferase | 0.6667 | -0.1815 | 0.0212 | -0.8739 |
| evm.TU.contig_2025.2 | threonyl-tRNA synthetase | 0.6282 | 0.3218 | 0.6485 | -0.1480 |
| evm.TU.contig_2273.8 | tyrosyl-tRNA synthetase | 0.0000 | -1.8598 | 0.0230 | -0.8878 |
| evm.TU.contig_590.2 | glutamyl-tRNA synthetase | 0.9315 | 0.0360 | 0.5711 | -0.1798 |
| evm.TU.contig_643.3 | lysyl-tRNA synthetase, class II | 0.0313 | 1.1223 | 0.8560 | -0.0772 |
| evm.TU.contig_2346.1 | glycyl-tRNA synthetase | 0.0060 | -0.8362 | 0.9954 | 0.0019 |
| evm.TU.contig_3583.2 | seryl-tRNA synthetase | 0.9859 | 0.0161 | 0.5627 | -0.3184 |
| evm.TU.contig_3558.3 | glycyl-tRNA synthetase | 0.7679 | 0.1343 | 0.0498 | 0.6650 |
| evm.TU.contig_4537.1 | aspartyl-tRNA synthetase | 0.0084 | 0.8427 | 0.0086 | 0.7244 |
| evm.TU.contig_2044.7 | leucyl-tRNA synthetase | 0.1081 | 0.5996 | 0.7797 | -0.1214 |
| evm.TU.contig_2113.8 | valyl-tRNA synthetase | 0.8838 | -0.0871 | 0.0214 | 0.7641 |
| evm.TU.contig_546.1 | isoleucyl-tRNA synthetase | 0.2723 | -0.5552 | 0.0109 | 0.6274 |
| evm.TU.contig_3384.26 | phenylalanyl-tRNA synthetase alpha chain | 0.1769 | -0.4357 | 0.0004 | -1.0194 |

**Table S9. Profiles of gene expression related to aminoacyl-tRNA biosynthesis (continued).**

| Gene ID | Annotation | FDR^1^ | Log_2_FC^1^ | FDR^2^ | Log_2_FC^2^ |
| --- | --- | --- | --- | --- | --- |
| evm.TU.contig_570.7 | tryptophanyl-tRNA synthetase | 0.1653 | -0.4798 | 0.0002 | -1.2098 |
| evm.TU.contig_2110.1 | seryl-tRNA synthetase | 0.8848 | -0.0867 | 0.0565 | 0.6404 |
| evm.TU.contig_2121.22 | methionyl-tRNA synthetase | 0.8522 | 0.0807 | 0.4437 | -0.2899 |
| evm.TU.contig_584.5 | glutaminyl-tRNA synthetase | 0.0050 | -1.1144 | 0.2215 | -0.4040 |
| evm.TU.contig_2025.28 | phenylalanyl-tRNA synthetase alpha chain | 0.2062 | 0.4878 | 0.1822 | -0.4292 |
| evm.TU.contig_766.1 | glutaminyl-tRNA synthetase | 0.8157 | -0.0880 | 0.1816 | -0.3580 |
| evm.TU.contig_2097.3 | leucyl-tRNA synthetase | 0.9012 | 0.1032 | 0.5760 | 0.3334 |
| evm.TU.contig_2288.12 | phenylalanyl-tRNA synthetase beta chain | 0.3641 | 0.4015 | 0.2365 | 0.4801 |
| evm.TU.contig_2306.5 | prolyl-tRNA synthetase | 0.0012 | -1.0770 | 0.0014 | -1.0235 |
| evm.TU.contig_3602.2 | prolyl-tRNA synthetase | 0.0131 | -0.9898 | 0.1209 | -0.4630 |
| evm.TU.contig_2421.10 | glutamyl-tRNA synthetase | 0.0063 | 1.0775 | 0.3166 | -0.2599 |
| evm.TU.contig_3464.2 | asparaginyl-tRNA synthetase | 0.0076 | -0.7427 | 0.0307 | -0.7984 |
| evm.TU.contig_2068.21 | histidyl-tRNA synthetase | 0.0019 | -1.0146 | 0.0012 | -1.4144 |
| evm.TU.contig_2286.9 | asparaginyl-tRNA synthetase | 0.0000 | -1.6654 | 0.0006 | -1.4645 |
| evm.TU.contig_2016.16 | valyl-tRNA synthetase | 0.8015 | -0.2151 | 0.6771 | -0.2001 |
| evm.TU.contig_4432.17 | methionyl-tRNA synthetase | 0.0049 | -1.0057 | 0.4557 | 0.2138 |
| evm.TU.contig_477.3 | cysteinyl-tRNA synthetase | 0.0064 | 1.0298 | 0.0086 | 0.8466 |

^1^ and ^2^ represent the differential expression on day 5 and day 15, respectively. Log_2_FC refers to the logarithm of the multiple of gene expression in the nitrogen-deficient group compared with that in the normal group.

**Table S10. Profiles of gene expression related to nitrogen metabolism**

| Gene ID | Annotation | FDR^1^ | Log_2_FC^1^ | FDR^2^ | Log_2_FC^2^ |
| --- | --- | --- | --- | --- | --- |
| evm.TU.contig_2305.7 | carbonic anhydrase | 0.0000 | -3.1163 | 0.0000 | -4.1033 |
| evm.TU.contig_943.1 | carbonic anhydrase | -- | -- | 0.4581 | -1.0638 |
| evm.TU.contig_2500.7 | carbonic anhydrase | 0.1039 | 0.4294 | 0.0294 | -0.5991 |
| evm.TU.contig_443.5 | carbonic anhydrase | 0.9217 | -0.1408 | 0.5119 | 1.0256 |
| evm.TU.contig_2055.1 | ferredoxin-nitrite reductase | 0.0000 | 2.8242 | 0.0000 | 6.3687 |
| evm.TU.contig_4581.1 | glutamate dehydrogenase (NAD(P)^+^) | 0.8190 | -0.1029 | 0.1186 | -1.1067 |
| evm.TU.contig_3428.3 | glutamate dehydrogenase (NADP^+^) | 0.0000 | -1.7966 | 0.4114 | -0.4791 |
| evm.TU.contig_4425.8 | glutamate dehydrogenase (NADP^+^) | 0.0079 | 1.2582 | 0.0107 | 0.7545 |
| evm.TU.contig_2294.1 | glutamine synthetase | 0.0145 | -1.2111 | 0.0001 | -1.3095 |
| evm.TU.contig_479.20 | glutamine synthetase | 0.0000 | 1.5502 | 0.0000 | 3.5664 |
| evm.TU.contig_3397.13 | MFS transporter, NNP family, nitrate/nitrite transporter | 0.0000 | 2.5560 | 0.0000 | 2.9101 |
| evm.TU.contig_2684.1 | MFS transporter, NNP family, nitrate/nitrite transporter | 0.0000 | 2.5415 | 0.0000 | 7.7007 |
| evm.TU.contig_2190.1 | MFS transporter, NNP family, nitrate/nitrite transporter | 0.0000 | 3.4022 | 0.0000 | 6.7014 |
| evm.TU.contig_2324.2 | MFS transporter, NNP family, nitrate/nitrite transporter | 0.0000 | 5.3142 | 0.0000 | 8.8473 |
| evm.TU.contig_3981.1 | MFS transporter, NNP family, nitrate/nitrite transporter | 0.0000 | 2.1579 | 0.0000 | 7.4385 |
| evm.TU.contig_2116.4 | nitrate reductase | 0.3905 | 0.3076 | 0.0001 | 1.0608 |
| evm.TU.contig_2324.1 | nitrate reductase (NAD(P)H) | 0.0000 | 3.6339 | 0.0000 | 7.9652 |
| evm.TU.contig_2190.2 | nitrate reductase (NAD(P)H) | 0.0550 | 0.6945 | 0.0000 | 5.2320 |

^1^ and ^2^ represent the differential expression on day 5 and day 15, respectively. Log_2_FC refers to the logarithm of the multiple of gene expression in the nitrogen-deficient group compared with that in the normal group.

**Table S11. Profiles of gene expression related to metabolism of fatty acids.**

| Gene ID | Annotation | FDR^1^ | Log_2_FC^1^ | FDR^2^ | Log_2_FC^2^ |
| --- | --- | --- | --- | --- | --- |
| evm.TU.contig_4490.1 | acetyl-CoA carboxylase / biotin carboxylase 1 | 0.0000 | -0.9176 | 0.0036 | -1.1826 |
| evm.TU.contig_3792.1 | [acyl-carrier-protein] S-malonyltransferase | 0.0000 | -1.8956 | 0.0146 | -1.0724 |
| evm.TU.contig_4451.6 | 3-oxoacyl-[acyl-carrier-protein] synthase II | 0.0032 | -0.7500 | 0.0000 | -1.5711 |
| evm.TU.contig_3410.10 | 3-oxoacyl-[acyl-carrier-protein] synthase II | 0.4461 | -0.3256 | 0.1308 | -0.5363 |
| evm.TU.contig_3503.3 | 3-oxoacyl-[acyl-carrier protein] reductase | 0.2038 | 0.8732 | 0.0654 | 1.1964 |
| evm.TU.contig_2102.13 | 3-oxoacyl-[acyl-carrier protein] reductase | 0.0000 | -6.2696 | 0.0000 | -6.4955 |
| evm.TU.contig_2117.4 | 3-oxoacyl-[acyl-carrier protein] reductase | 0.0000 | 2.7958 | 0.1849 | -1.0690 |
| evm.TU.contig_465.1 | 3-oxoacyl-[acyl-carrier protein] reductase | 0.3957 | -0.2417 | 0.0022 | 1.3242 |
| evm.TU.contig_456.10 | 3-hydroxyacyl-[acyl-carrier-protein] dehydratase | 0.5878 | -0.2799 | 0.0003 | -1.7299 |
| evm.TU.contig_3606.4 | enoyl-[acyl-carrier protein] reductase I | 0.0000 | -2.2782 | 0.0003 | -1.4299 |
| evm.TU.contig_2273.12 | long-chain-fatty-acid--CoA ligase ACSBG | 0.0367 | -0.9015 | 0.5313 | -0.3448 |
| evm.TU.contig_435.8 | long-chain-fatty-acid--CoA ligase ACSBG | 0.0000 | 1.6223 | 0.0000 | 4.7463 |
| evm.TU.contig_2696.2 | long-chain acyl-CoA synthetase | 0.6749 | -0.3663 | 0.3434 | -0.4083 |
| evm.TU.contig_516.13 | long-chain acyl-CoA synthetase | 0.9714 | -0.0190 | 0.0084 | 0.9798 |
| evm.TU.contig_2082.7 | long-chain acyl-CoA synthetase | 0.0016 | 1.1929 | 0.0000 | -1.6467 |
| evm.TU.contig_3401.11 | very-long-chain (3R)-3-hydroxyacyl-CoA dehydratase | 0.1514 | 0.4058 | 0.0242 | 0.8125 |
| evm.TU.contig_2016.10 | very-long-chain (3R)-3-hydroxyacyl-CoA dehydratase | 0.7476 | -0.1256 | 0.0252 | -0.8224 |
| evm.TU.contig_491.2 | 17beta-estradiol 17-dehydrogenase/very-long-chain 3-oxoacyl-CoA reductase | 0.5093 | -0.3860 | 0.8766 | 0.0708 |

**Table S11. Profiles of gene expression related to metabolism of fatty acids (continued).**

| Gene ID | Annotation | FDR^1^ | Log_2_FC^1^ | FDR^2^ | Log_2_FC^2^ |
| --- | --- | --- | --- | --- | --- |
| evm.TU.contig_3572.1 | fatty acid elongase 3 | 0.0059 | 0.6492 | 0.0867 | 0.8123 |
| evm.TU.contig_3397.10 | 3-ketoacyl-CoA synthase | 0.0096 | 0.7700 | 0.4303 | 0.4757 |
| evm.TU.contig_700.1 | acyl-CoA oxidase | 0.0000 | -2.3212 | 0.8668 | 0.0985 |
| evm.TU.contig_545.4 | acyl-CoA oxidase | 0.0000 | -2.2805 | 0.3517 | -0.3187 |
| evm.TU.contig_3399.4 | acyl-CoA oxidase | 0.4385 | -0.2350 | 0.0013 | -1.2461 |
| evm.TU.contig_2025.12 | enoyl-CoA hydratase/3-hydroxyacyl-CoA dehydrogenase | 0.0002 | -0.7975 | 0.5153 | -0.3029 |
| evm.TU.contig_2062.16 | acetyl-CoA C-acetyltransferase | 0.7578 | -0.1265 | 0.3966 | -0.2889 |
| evm.TU.contig_2285.10 | acetyl-CoA C-acetyltransferase | 0.0001 | -0.9293 | 0.0001 | -1.4215 |
| evm.TU.contig_606.9 | 3,2-trans-enoyl-CoA isomerase, mitochondrial | 0.4062 | 0.2680 | 0.6578 | 0.1644 |
| evm.TU.contig_2292.7 | aldehyde dehydrogenase (NAD^+^) | 0.6930 | 0.1639 | 0.9535 | -0.0251 |
| evm.TU.contig_563.3 | aldehyde dehydrogenase (NAD^+^) | 0.9968 | -0.0061 | 0.9394 | 0.1361 |
| evm.TU.contig_563.2 | aldehyde dehydrogenase (NAD^+^) | 0.6416 | -0.6000 | 0.6164 | -0.6595 |
| evm.TU.contig_456.3 | aldehyde dehydrogenase family 7 member A1 | 0.0026 | -1.1714 | 0.0000 | -1.2835 |
| evm.TU.contig_4516.3 | S-(hydroxymethyl)glutathione dehydrogenase/alcohol dehydrogenase | 0.0176 | -1.0571 | 0.0000 | -2.6577 |

^1^ and ^2^ represent the differential expression on day 5 and day 15, respectively. Log_2_FC refers to the logarithm of the multiple of gene expression in the nitrogen-deficient group compared with that in the normal group.

**Figure S1. Changes of F_v_/F_m_ during growth.**

**Figure S2. Changes of chlorophyll *a* content during growth.**

**Figure S3.** **Transcriptome annotation in various databases.**

**Figure S4. COG function classification of all genes.**


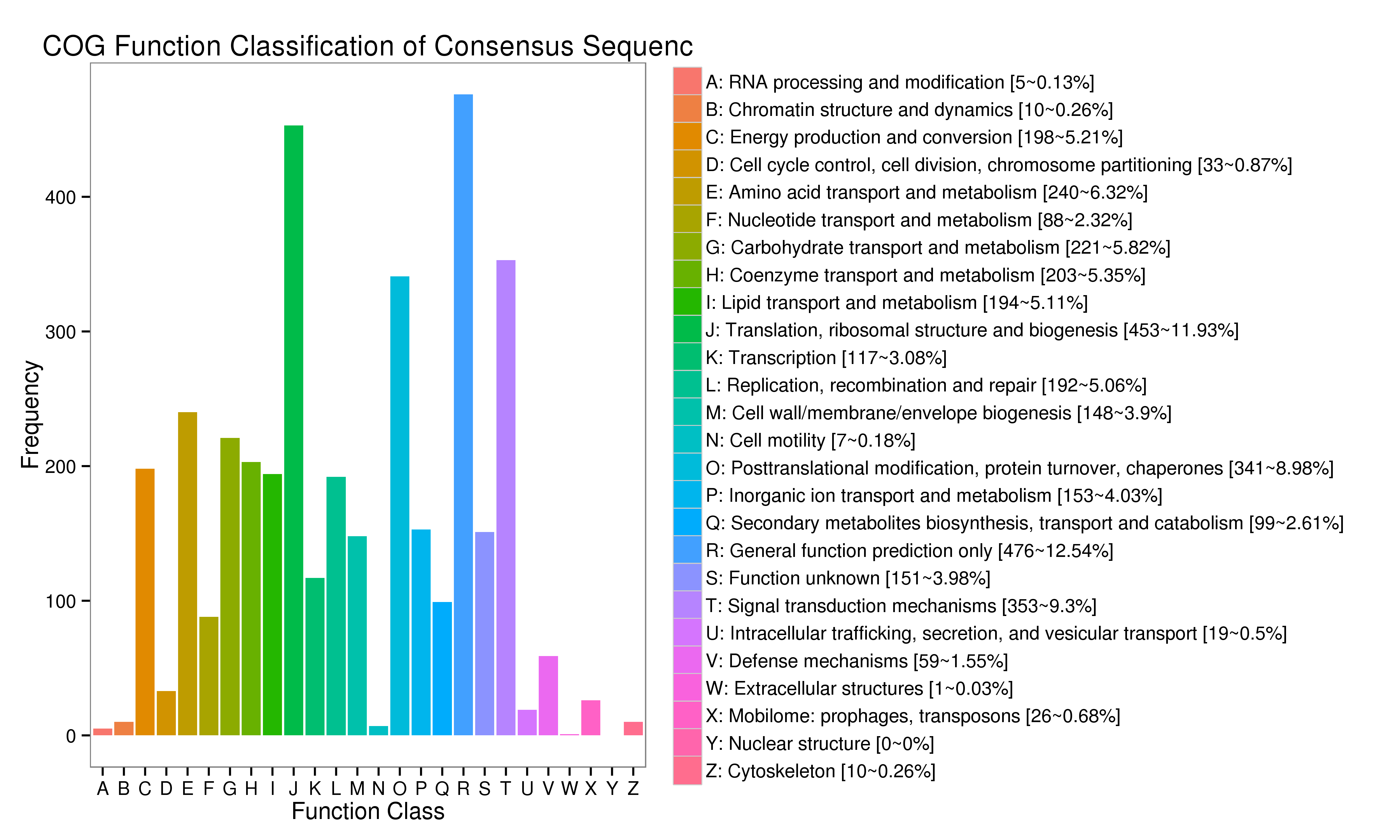


**Figure S5.** **GO function classification of all genes.**


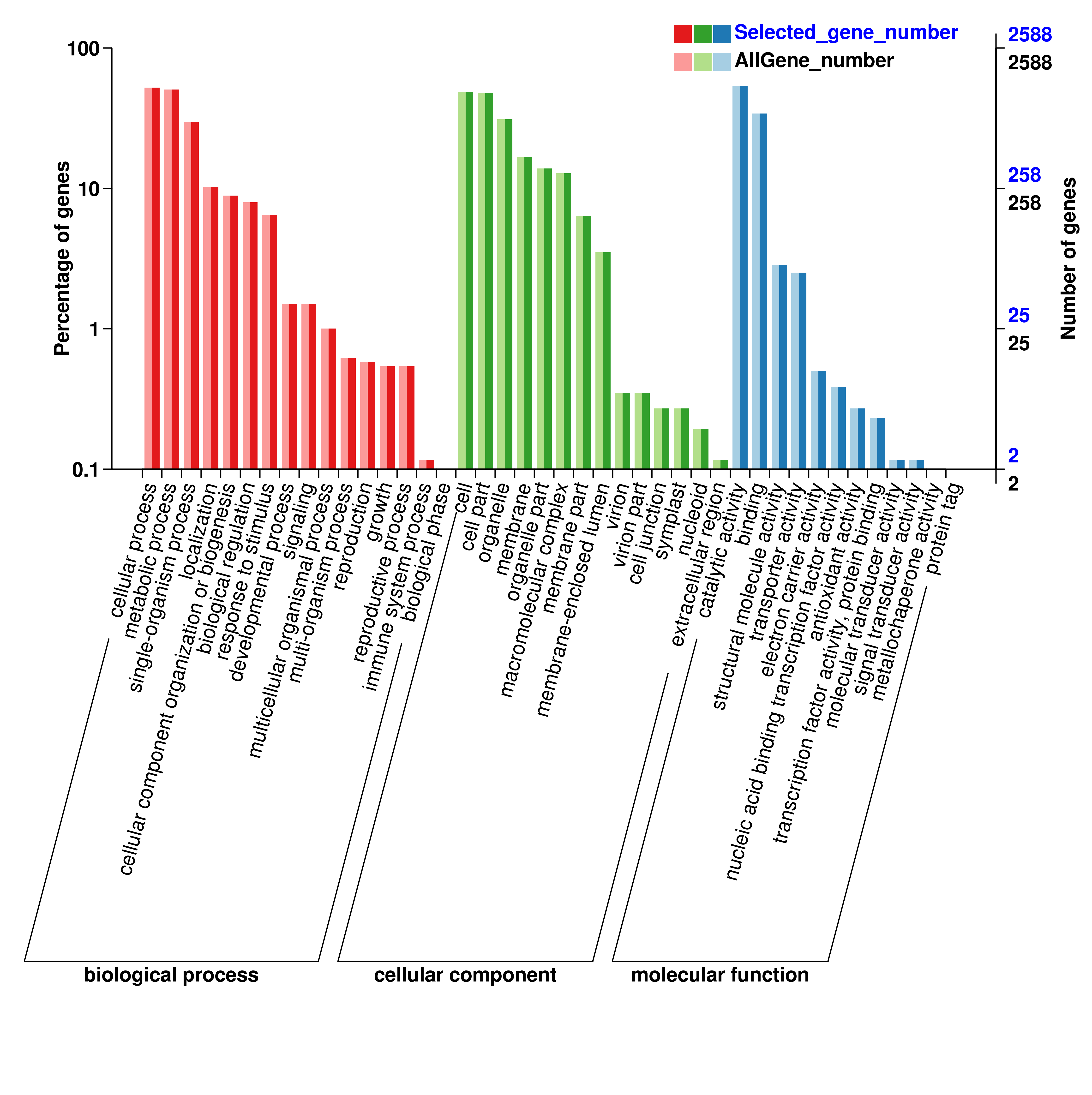


**Figure S6.** **KEGG function classification of all genes.**


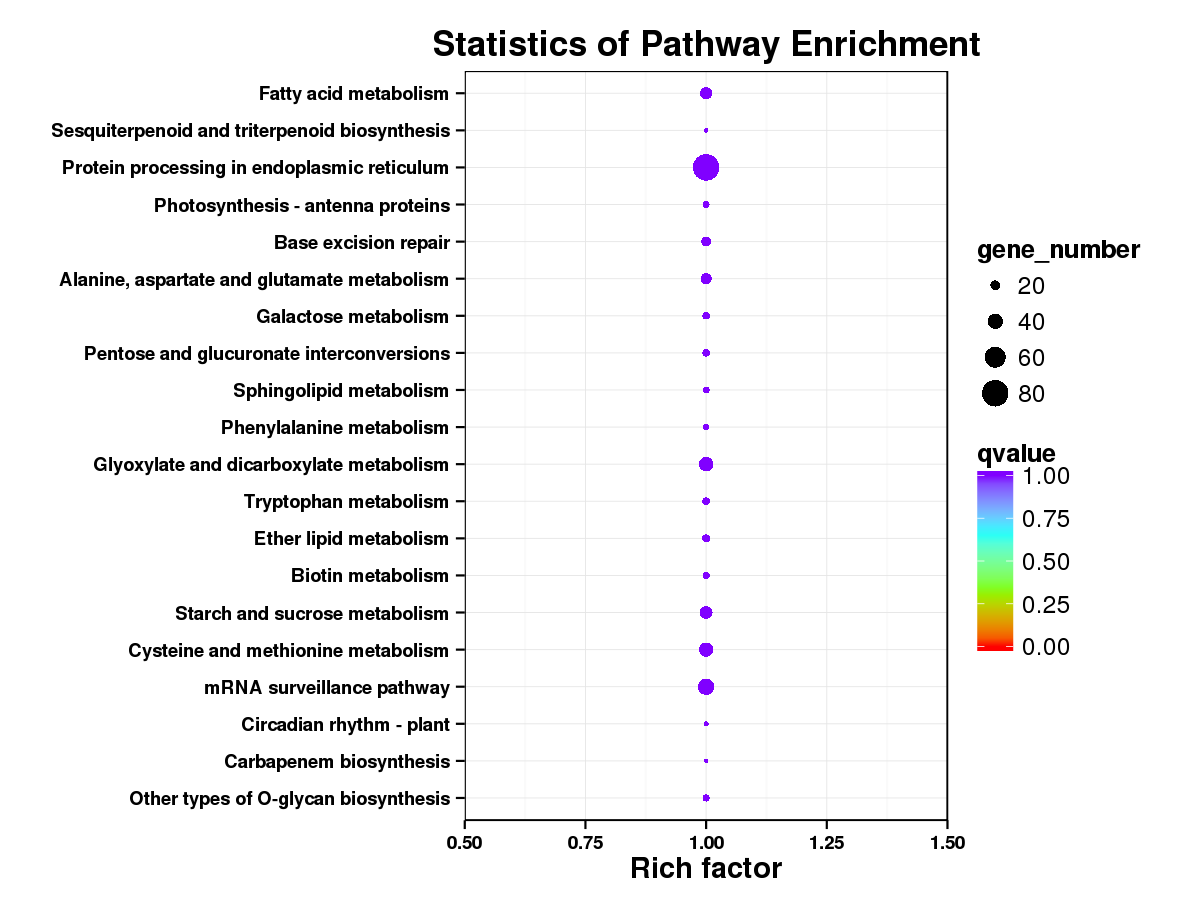


**Figure S7.** **COG function classification of DEGs in R15_vs_Y15.**

**
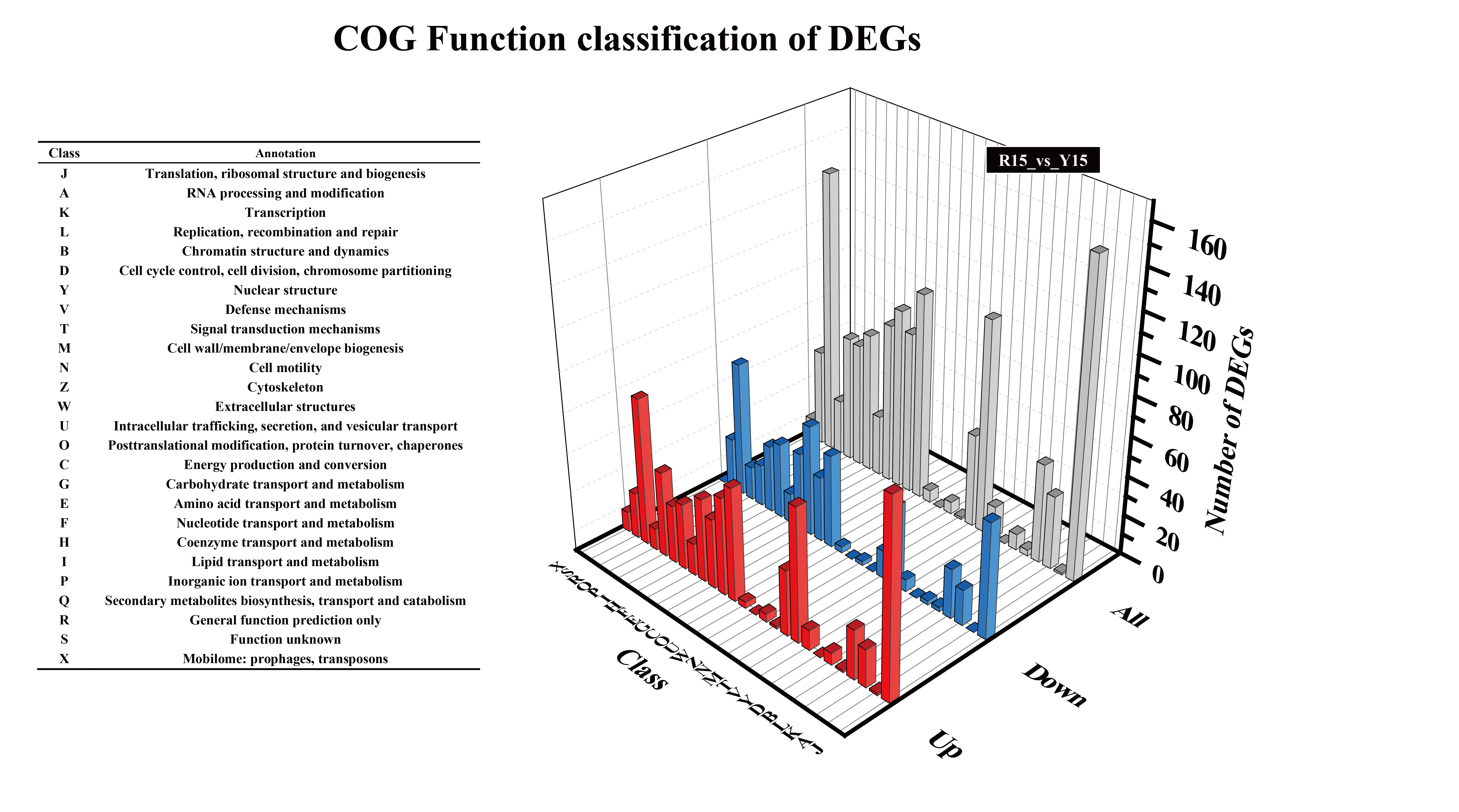
**

**Figure S8.** **GO function classification of DEGs in R5_vs_Y5.**

**
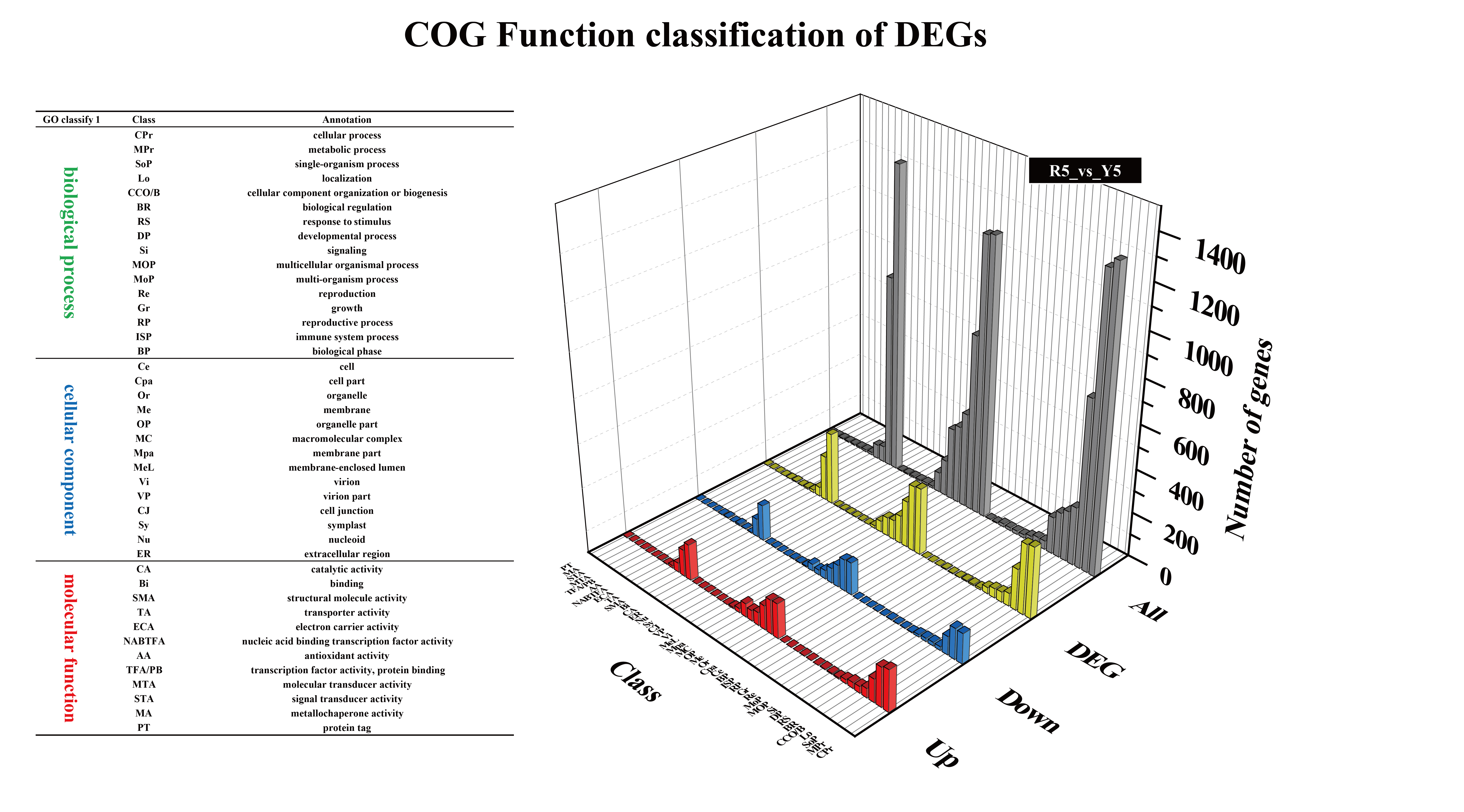
**

**Figure S9.** **GO function classification of DEGs in R15_vs_Y15.**

**
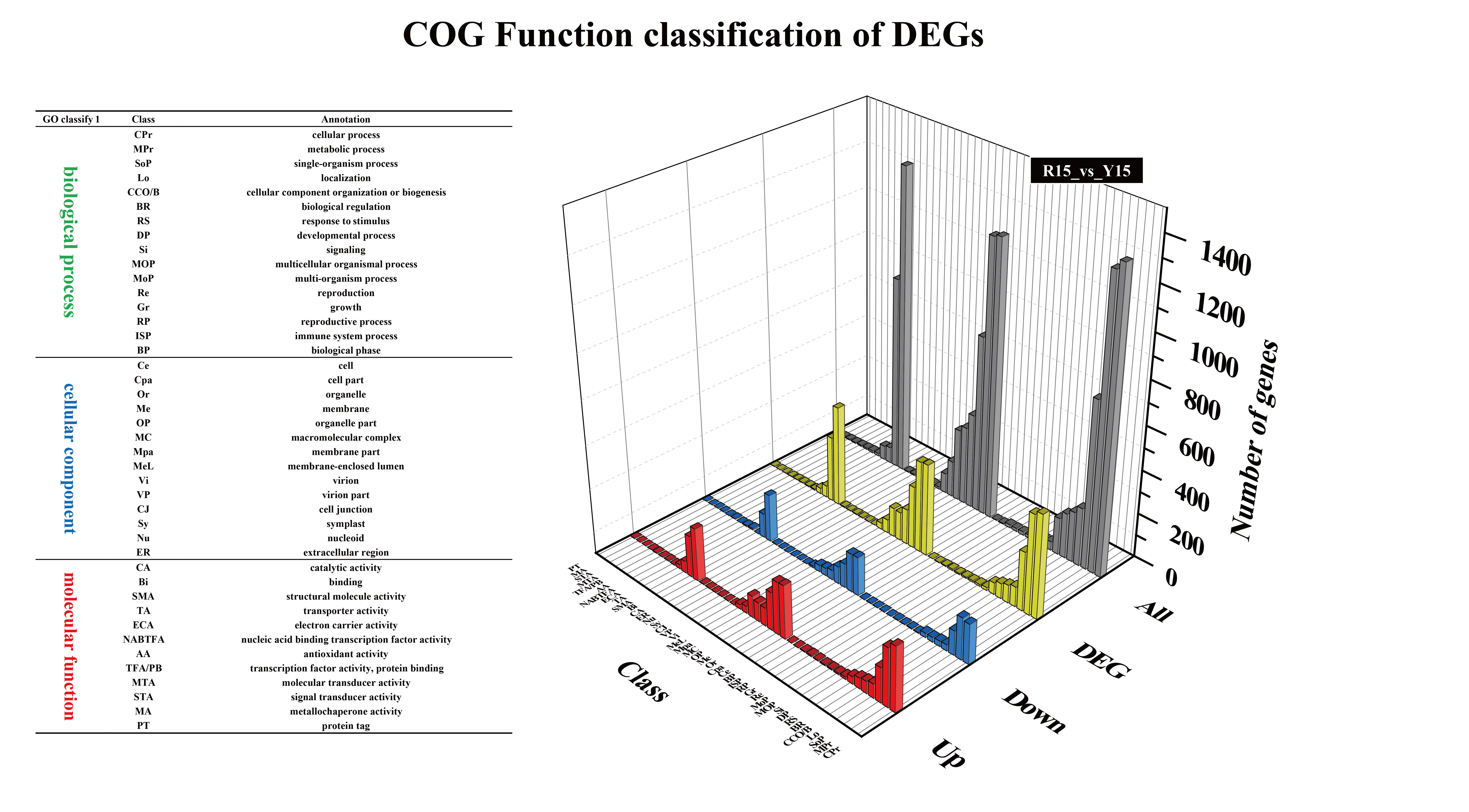
**

**Figure S10. KEGG function classification of DEGs in R5_vs_Y5.**

**
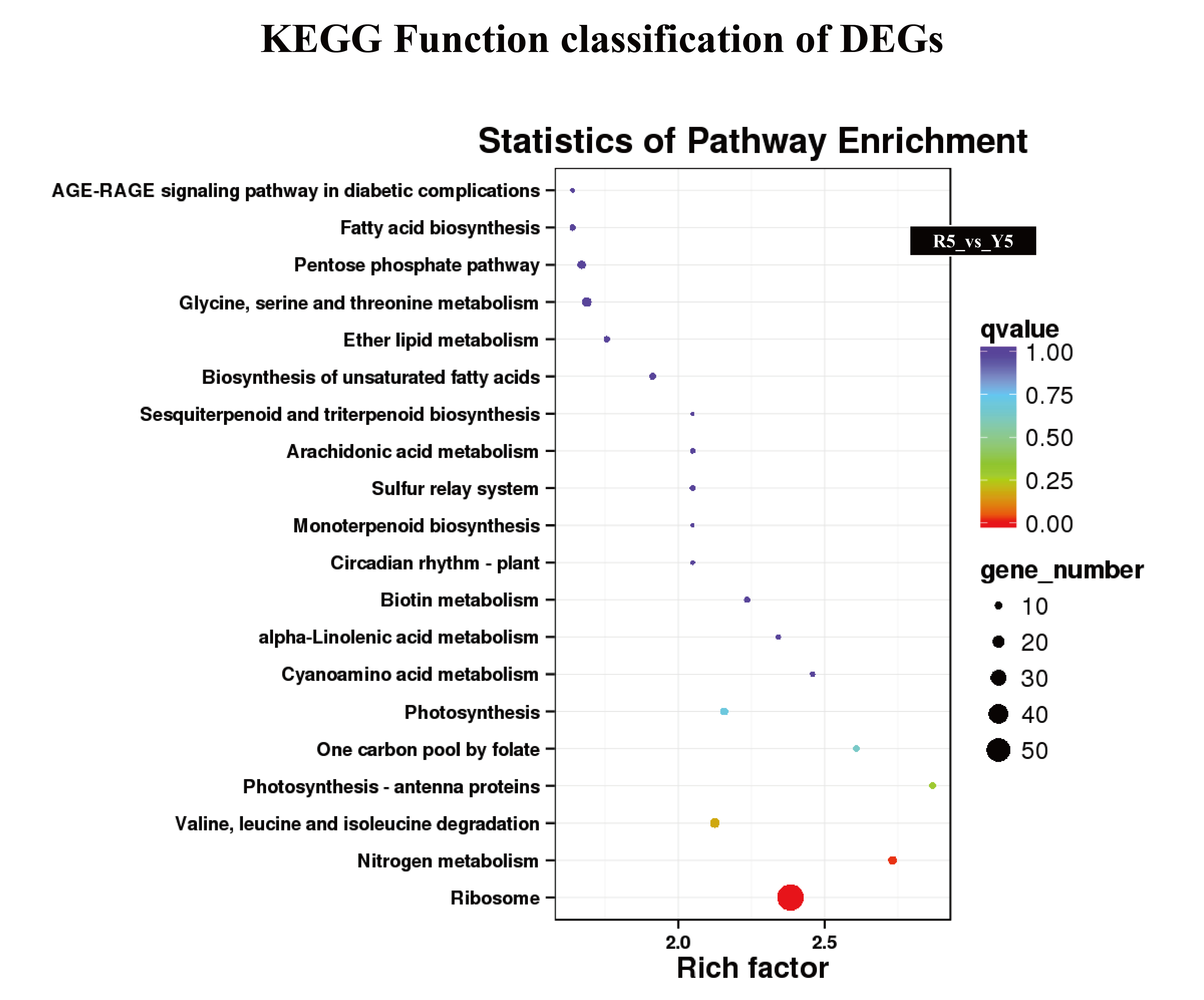
**

**Figure S11.** **KEGG function classification of DEGs in R15_vs_Y15.**

**
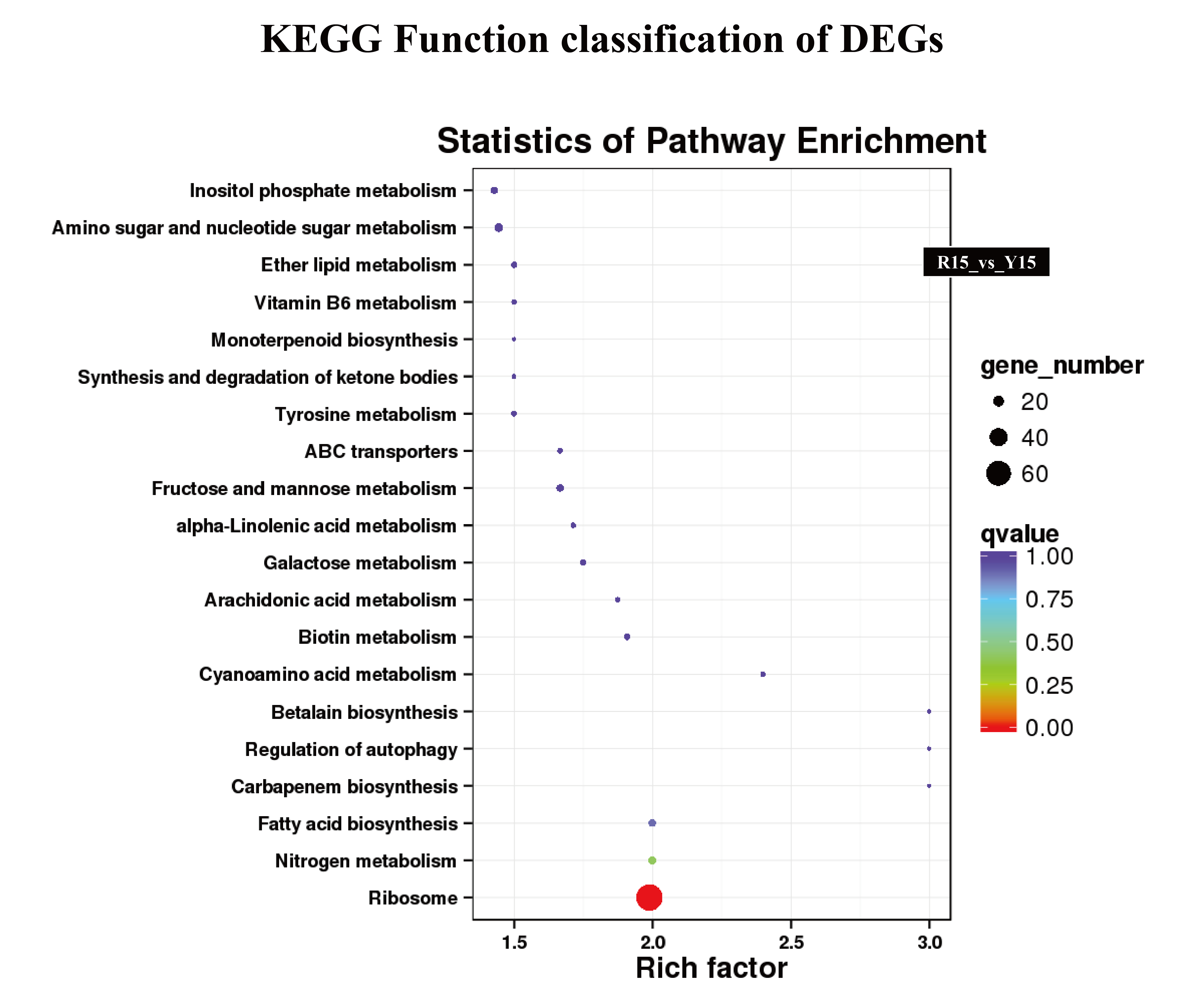
**

**Figure S12. GC-MS information of C18:3(9,12,15).**


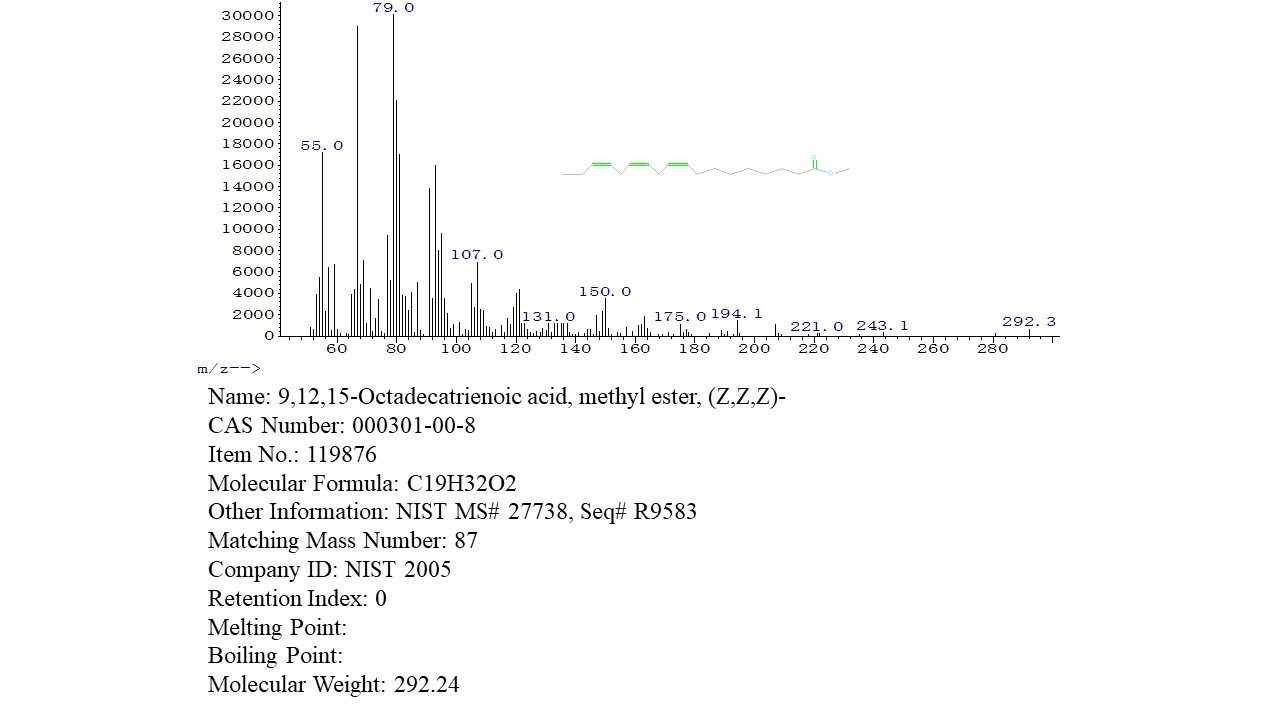


**Figure S13.** **GC-MS information of C18:3(6,9,12).**


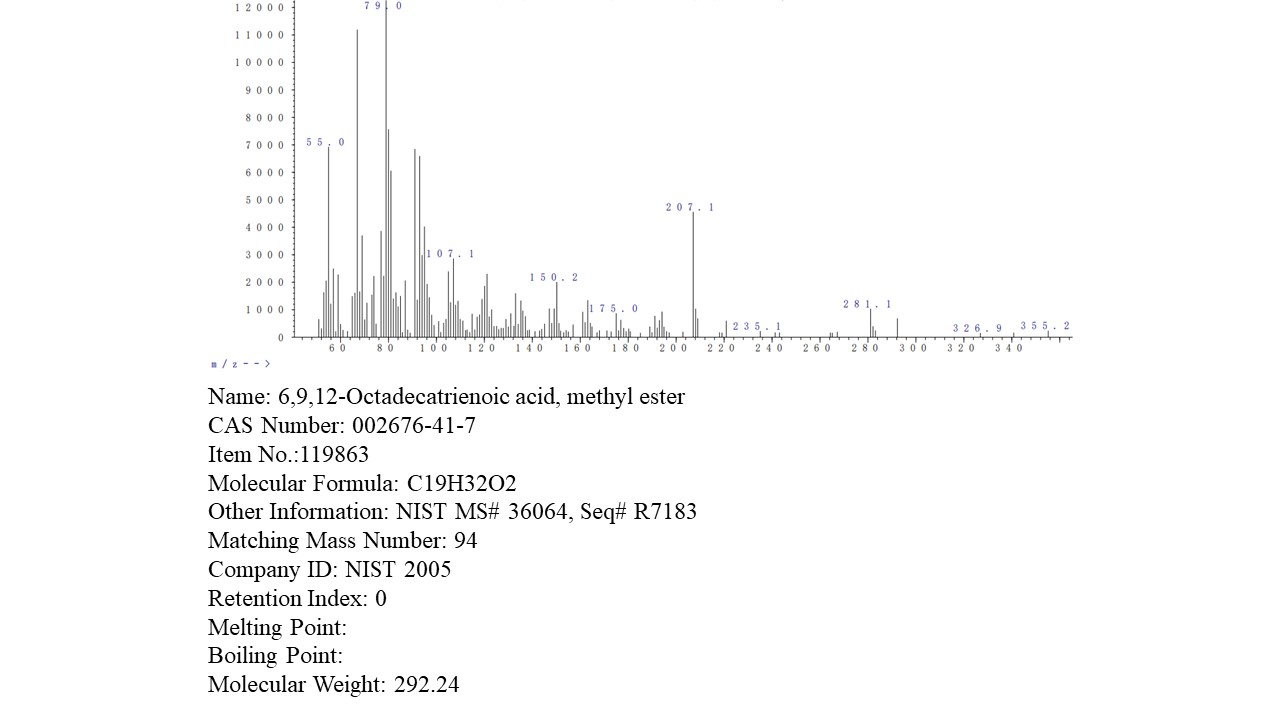


C18:3(6,9,12) was detected in our previous experiment, and the inconsistent peak times were due to different experimental parameters.

**Figure S14.** **GC-MS information of C20:3(11,14).**


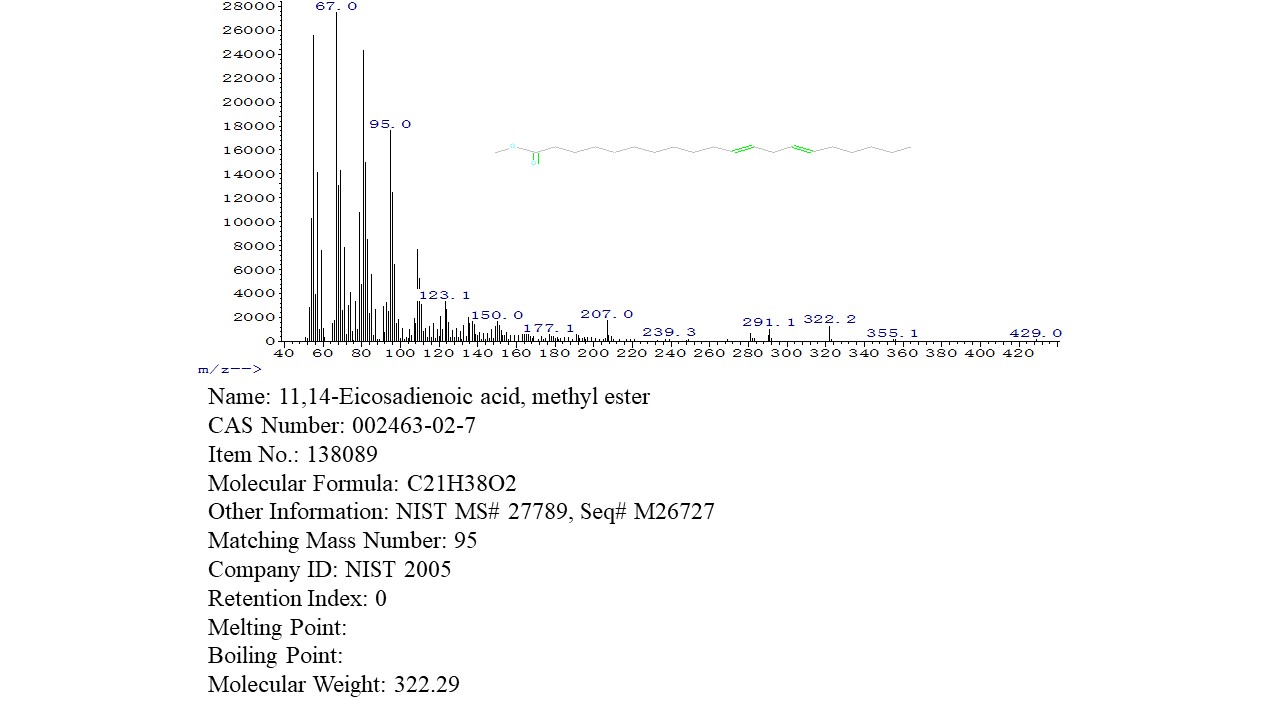

Supplement: Supplementary file 1 — Additional file 1: Table S1. Summary of reads in control and nitrogen deprivation of P. purpureum transcriptomes. Table S2. Profiles of gene expression related to photosynthesis. Table S3. Profiles of gene expression related to oxidative phosphorylation. Table S4. Profiles of gene expression related to pentose phosphate pathway. Table S5. Profiles of gene expression related to carbon fixation. Table S6. Profiles of gene expression related to gluconeogenesis and glycolysis. Table S7. Profiles of gene expression related to TCA cycle. Table S8. Profiles of gene expression related to ubiquitin mediated proteolysis. Table S9. Profiles of gene expression related to aminoacyl-tRNA biosynthesis. Table S10. Profiles of gene expression related to nitrogen metabolism. Table S11. Profiles of gene expression related to metabolism of fatty acids. Figure S1. Changes of Fv/Fm during growth. Figure S2. Changes of chlorophyll a content during growth. Figure S3. Transcriptome annotation in various databases. Figure S4. COG function classification of all genes. Figure S5. GO function classification of all genes. Figure S6. KEGG function classification of all genes. Figure S7. COG function classification of DEGs in R15_vs_Y15. Figure S8. GO function classification of DEGs in R5_vs_Y5. Figure S9. GO function classification of DEGs in R15_vs_Y15. Figure S10. KEGG function classification of DEGs in R5_vs_Y5. Figure S11. KEGG function classification of DEGs in R15_vs_Y15. Figure S12. GC-MS information of C18:3(9,12,15). Figure S13. GC-MS information of C18:3(6,9,12). Figure S14. GC-MS information of C20:3(11,14). [file 40643_2021_426_MOESM1_ESM.docx]
